# Development and validation of a digital PCR assay targeting plasma *FAR1* methylation for early detection of hepatocellular carcinoma

**DOI:** 10.1080/15592294.2026.2653956

**Published:** 2026-04-20

**Authors:** Sun Jae Park, Yun Young Lee, Yong-Kyu Chung, Bo-Hyun Jung, Joon An, Jinil Han, Youngho Moon, Hee-Jung Wang

**Affiliations:** a Department of Surgery, Inje University Haeundae Paik Hospital, Busan, Republic of Korea; b R&D Center, Gencurix, Inc, Seoul., Republic of Korea

**Keywords:** Hepatocellular carcinoma, early diagnosis, DNA methylation, digital PCR, circulating tumor DNA, biomarkers

## Abstract

Hepatocellular carcinoma (HCC) is a leading cause of cancer-related mortality worldwide, yet current surveillance with alpha-fetoprotein (AFP) and ultrasound (US) lacks sufficient accuracy, particularly for early-stage disease. This study aimed to develop and clinically validate a blood-based digital PCR (dPCR) assay targeting novel methylation biomarkers for HCC detection. Genome-wide methylation profiles from HCC, normal, and other cancer types were analysed to identify candidates, which were then screened and verified in cancer cell lines, primary tumour tissues, and plasma specimens. A total of 186 plasma samples were evaluated, including 66 healthy controls, 60 patients with chronic liver disease (CLD) without HCC, and 60 patients with HCC. *FAR1* methylation emerged as an HCC-specific biomarker, showing significantly higher levels in liver cancer cell lines and HCC tumour tissues compared with controls. A dPCR assay targeting *FAR1* achieved 70.0% sensitivity (42/60; 95% CI, 56.8%–81.2%) and 96.8% specificity (122/126; 95% CI, 92.1–99.1%), with an area under the curve (AUC) of 0.90 (95% CI, 0.85–0.96). Combining *FAR1* with AFP improved sensitivity to 88.3% (53/60; 95% CI, 77.4%–95.2%) while maintaining 96.8% specificity. Notably, early-stage HCC (Barcelona Clinic Liver Cancer [BCLC] stage 0–A) was detected with 85.4% sensitivity (35/41; 95% CI, 77.4%–94.4%). This blood-based dPCR assay demonstrated high diagnostic performance, underscoring its potential as a noninvasive tool to enhance early detection and clinical management of HCC in high-risk populations.

## Introduction

Liver cancer is the third leading cause of cancer-related mortality worldwide, accounting for over 750,000 deaths in 2022 [1]. Hepatocellular carcinoma (HCC), the most common form of primary liver cancer, is associated with poor prognosis due to its frequent diagnosis at advanced stages, when curative options are limited [2]. The global incidence of HCC continues to rise, particularly in regions with high prevalence of hepatitis B or C virus infections and increasing rates of metabolic dysfunction – associated steatotic liver disease (MASLD) [3]. Despite therapeutic advances – including surgery, liver transplantation, and targeted therapies – overall survival remains low, emphasizing the need for improved early detection strategies.

Current guidelines recommend HCC surveillance with ultrasound (US) and alpha-fetoprotein (AFP) every six months for high-risk individuals, such as those with chronic liver disease (CLD) or cirrhosis [4]. However, US shows limited sensitivity for early-stage tumours, particularly in obese patients or those with cirrhotic liver morphology, while AFP lacks adequate specificity and often fails to detect early HCC [5–8]. Even in combination, their sensitivity remains approximately 60% [9]. Although computed tomography (CT) and magnetic resonance imaging (MRI) may improve detection, their widespread use is constrained by cost, accessibility, and, for CT, radiation exposure [10]. Accordingly, novel noninvasive biomarkers with improved accuracy are urgently needed to enable early diagnosis and expand access to curative interventions.

In response, various biomarker-based strategies have been investigated for HCC surveillance. The GALAD model, which incorporates demographic and serum protein markers – including AFP, AFP-L3 (lens culinaris agglutinin-reactive fraction of AFP), and DCP (des-gamma-carboxy prothrombin) – achieved an AUC (area under the curve) of 0.78 in multicenter prospective studies [11,12]. Methylation-based assays, such as the serum *SEPT9* test (AUC = 0.81) and a multi-target blood test combining three methylated DNA markers with AFP (AUC = 0.92), have also demonstrated promising diagnostic performance [13,14]. Multi-analyte panels that integrate protein and epigenetic biomarkers have been evaluated to further enhance diagnostic accuracy. However, despite these advances, no biomarker-based assay has yet been incorporated into routine clinical practice, underscoring the need for further validation.

Against this background, this study aimed to develop a novel blood-based digital PCR (dPCR) assay targeting tumour-specific DNA methylation markers for the early detection of HCC. Candidate biomarkers were identified through large-scale methylome analysis, optimized for quantitative detection in plasma-derived circulating tumour DNA (ctDNA), and clinically validated against AFP to assess diagnostic performance. This approach may provide a sensitive and specific diagnostic tool to improve early detection and facilitate routine clinical implementation in HCC surveillance.

## Materials and methods

### Study design

**Figure 1.**
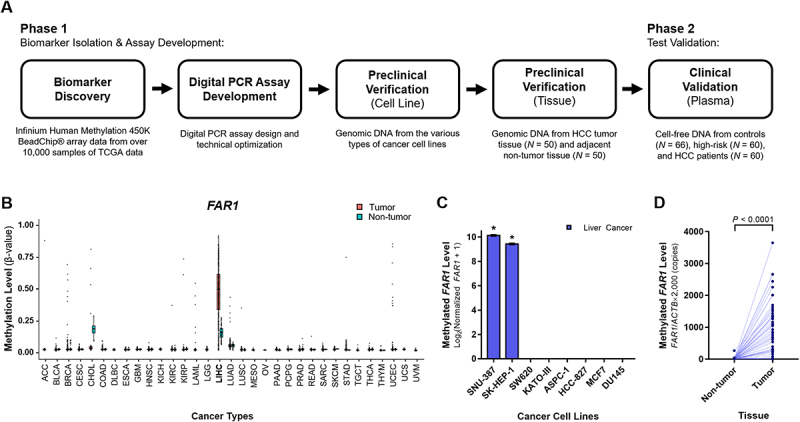
Development of a diagnostic tool for hepatocellular carcinoma (HCC) using novel HCC-specific DNA methylation biomarkers. (a) Schematic representation of the study workflow. (b) DNA methylation levels of *FAR1* across 33 cancer types and normal tissues. (C) *FAR1* methylation status in eight cancer cell lines, including two liver cancer cell lines (SNU-387 and SK-HEP-1), were analyzed. Cancer cell lines from colon (SW620), stomach (KATO-III), pancreas (ASPC-1), lung (HCC-827), breast (MCF7), and prostate (DU145) were also analyzed. Digital PCR results are presented as means ± sd from three independent experiments. Differences were assessed using one-way ANOVA followed by Tukey’s multiple comparisons test (*p < 0.0001). (d) *FAR1* methylation levels were analyzed in primary tumors and paired adjacent non-tumor tissues from 50 patients with HCC. Samples from the same patient are connected by a line. Differences were assessed using paired *t*-test.

This study was conducted in two phases: 1) biomarker discovery and 2) clinical validation (Figure 1(A)). Candidate methylation biomarkers were identified, developed into a dPCR assay, and validated in a retrospective case-control study using plasma specimens collected between January 2019 and October 2022. A total of 186 plasma samples were analysed, comprising 66 healthy controls, 60 patients with CLD without HCC, and 60 patients with HCC (68% of whom had early-stage disease). Inclusion criteria were age ≥ 19 years, availability of clinical data, and sufficient plasma volume for cell-free DNA (cfDNA) extraction. Exclusion criteria included prior systemic therapy, recurrent HCC, other malignancies, inadequate sample quality, and hemolyzed specimens. The target sample size of 170 was determined a priori based on a superiority test for sensitivity and specificity. Assuming a null hypothesis of 76% sensitivity and 81% specificity, with an expected sensitivity of 89% and specificity of 90%, a minimum of 60 HCC cases and 110 non-cancer samples were required to achieve 80% power at a two-sided α of 0.05. The final cohort size slightly exceeded this number based on sample availability. The primary outcome was the sensitivity and specificity in distinguishing HCC from high-risk individuals and healthy controls. Secondary outcomes included sensitivity for early-stage HCC, as well as positive and negative predictive values (PPV and NPV).

### Patient information

The use of all human-derived tissue and plasma samples was approved for exemption from review by the Public Institutional Review Board designated by the Ministry of Health and Welfare of Korea. The requirement for written informed consent was waived due to the retrospective nature of the study, minimal risk and the use of anonymized data. All procedures complied with the Declaration of Helsinki. For tissue-based analysis, frozen primary tumour and matched non-tumour tissues from 50 HCC patients were obtained from the Biobank of Ajou University Hospital (P01-202001-31–005); clinical characteristics are summarized in Supplemental Table S1. For clinical validation, plasma samples were collected from healthy controls, CLD patients without HCC, and HCC patients (P01-202312-02–010). As shown in Table 1, HCC cases were enriched for early-stage disease and categorized using the Barcelona Clinical Liver Cancer (BCLC) staging system. To further evaluate assay specificity, plasma from individuals with hepatitis B, hepatitis C, MASLD, or alcoholic steatohepatitis (ASH), as well as from asymptomatic healthy donors, was included. All samples met institutional quality-control standards.

**Table 1.** Characteristics and demographics of the validation cohort.

Characteristics	HCC^a^	Non-HCC	
		High-risk^a^	Healthy Controls^b^
**Total**, *n*	60	60	66
**Sex**, *n* **(%)**			
Male	52 (86.7)	39 (65.0)	25 (37.9)
Female	8 (13.3)	21 (35.0)	41 (62.1)
**Age (years) – Median (range)**	59.5 (45–86)	48.0 (23–80)	49.0 (21–73)
**Chronic Liver Diseases**, *n* **(%)**			
HBV	34 (56.7)	15 (25.0)	–
HCV	6 (10.0)	15 (25.0)	–
MASLD	0 (0.0)	15 (25.0)	–
ASH	14 (23.3)	15 (25.0)	–
Cirrhosis	49 (81.7)	0 (0.0)	–
Unknown	8 (13.3)	0 (0.0)	–
**Child-Pugh Score**, *n* **(%)**			
A	50 (83.4)	17 (28.3)	–
B	6 (10.0)	0 (0.0)	–
C	2 (3.3)	0 (0.0)	–
Unknown	2 (3.3)	43 (71.7)	–
**BCLC Stage**, *n* **(%)**			
0	18 (30.0)	–	–
A	23 (38.3)	–	–
B	6 (10.0)	–	–
C	13 (21.7)	–	–

^a^Plasma obtained from the Ajou University Hospital (Suwon, Republic of Korea).

^b^40 plasma samples purchased from Innovative Research (Novi, MI, USA), and 26 specimens obtained from the Ajou University Hospital (Suwon, Republic of Korea).

–, No patients in category.

HCC, hepatocellular carcinoma; HBV, hepatitis B virus; HCV, hepatitis C virus; MASLD, metabolic dysfunction-associated steatotic liver disease; ASH, alcoholic steatohepatitis; BCLC, Barcelona Clinical Liver Cancer.

### Biomarker discovery and verification

DNA methylation profiles from 10,000 tissue samples – including HCC, normal, and other cancer types – were analysed using the ChAMP package for β-value extraction and normalization [15,16]. Public datasets from The Cancer Genome Atlas (TCGA), generated using the Infinium Human Methylation 450K BeadChip array (Illumina, San Diego, CA, USA) were utilized. Candidate CpGs were selected based on four criteria: (1) low methylation in blood cells; (2) high methylation in HCC tumours compared with adjacent normal liver tissues; (3) low methylation in other cancer types; and (4) low methylation in normal tissues. These criteria were applied through a stepwise filtering pipeline starting from genome-wide methylation array data, followed by successive exclusion based on predefined thresholds in blood cells (median β-value > 0.1), HCC tissues (median β-value < 0.5), and non-HCC tissues (median β-value > 0.2). Candidate CpGs were then ranked according to sensitivity and specificity for HCC detection, and further narrowed down through digital PCR assay design, cell line verification, tissue validation, and plasma-based testing, ultimately resulting in three methylation biomarkers. Importantly, final marker selection prioritized CpGs that demonstrated robust analytical performance in dPCR and consistent detectability in plasma, as these characteristics are essential for reliable liquid biopsy-based clinical application. Oligonucleotide sets targeting selected regions were designed and optimized for dPCR. Genomic regions covered by primers and probes (Integrated DNA Technologies, Coralville, IA, USA) are listed in Supplemental Table S2. Assay conditions were validated using Human HCT116 DKO Non-methylated and Methylated DNA (Zymo Research, Irvine, CA, USA) as standards [17,18], and further verified using DNA extracted from human cancer cell lines (Korean Cell Line Bank, Seoul, Republic of Korea) and tissue samples from the Biobank of Ajou University Hospital (Suwon, Republic of Korea). DNA was extracted using the Wizard Genomic DNA Purification Kit (Promega, Madison, WI, USA), quantified with a NanoDrop 2000 Spectrophotometer (Thermo Scientific, Waltham, MA, USA), and bisulphite-converted using the EZ DNA Methylation-Lightning Kit (Zymo Research). The final product was analysed by dPCR according to standard protocols.

### Plasma-based assay validation

Peripheral blood was collected in BD Vacutainer K2EDTA tubes (Becton Dickinson, Franklin Lakes, NJ, USA) prior to treatment. Plasma was separated within 4 hours by double centrifugation at 3,000 × g for 10 minutes and stored at −70°C. cfDNA was extracted from 1.5–2.0 mL of plasma using the QIAamp Circulating Nucleic Acid Kit (Qiagen, Hilden, Germany), then bisulphite-converted using the EZ DNA Methylation-Lightning Kit (Zymo Research) according to the manufacturer’s protocols. Bisulphite-treated DNA was analysed using the Q×600 Droplet Digital PCR System (Bio-Rad Laboratories, Hercules, CA, USA). Reaction mixtures contained 10 μL of template DNA, 3.3 μL of 10 μM Oligo Mix (Gencurix, Inc., Seoul, Republic of Korea), 5.5 μL of 4X Droplet Digital PCR Multiplex Supermix, and 0.33 μL of 0.3 M dithiothreitol, in a total volume of 22 μL. Droplets were generated using the QXDx Droplet Generator and amplified using a Veriti 96-Well Thermal Cycler (Applied Biosystems, Foster City, CA, USA) under the following conditions: 95°C for 10 minutes; 45 cycles of 94°C for 30 seconds and 59°C for 1 minute; and 98°C for 10 minutes. Droplets were read using the Q×600 Droplet Digital PCR Reader and analysed with QXManager version 2.1 software. Each run included no-template and positive controls. Droplet classification thresholds were determined automatically by the instrument software based on fluorescence amplitude distribution. Briefly, positive control wells were used to define baseline and target droplet clusters, and the threshold was set at the midpoint between the median fluorescence amplitudes of the negative (baseline) and positive (target) droplet populations. This threshold was uniformly applied to all samples within the same run, and target copy numbers were calculated from the number of positive droplets using Poisson statistics. Results were expressed as copies/mL, with *ACTB* used as an internal control. All procedures followed the dMIQE (Minimum Information for Publication of Quantitative Digital PCR Experiments) guidelines [18].

### Statistical analysis

All statistical analyses were performed using GraphPad version 7 (GraphPad Software, La Jolla, CA, USA) and MedCalc version 15.8 (MedCalc, Ostend, Belgium). Receiver-operating characteristic (ROC) curve analysis was used to evaluate sensitivity, specificity, and AUC, with 95% confidence intervals (CIs) calculated for all clinical performance metrics. Group differences were assessed using one-way ANOVA followed by Tukey’s multiple comparison test. The Mann-Whitney U test was applied to compare methylation levels according to clinicopathological variables. A *p*-value < 0.05 was considered statistically significant. For evaluation of combined biomarker performance, a multivariate logistic regression model including plasma *FAR1* methylation and serum AFP levels was constructed to estimate the probability of HCC, and model performance was assessed using ROC curve analysis. In addition, to assess potential confounding effects, a separate multivariable logistic regression model including plasma *FAR1* methylation and sex was constructed, and odds ratios with 95% CIs were calculated.

## Results

This study consisted of two phases: biomarker discovery and assay development (Phase 1), followed by validation in a retrospective case-control plasma cohort (Figure 1(A)).

### Discovery and characterization of novel biomarkers

DNA methylation profiles from over 10,000 tissue samples in public datasets generated using the Infinium Human Methylation 450K BeadChip array were analysed to identify candidate biomarkers for early HCC detection. Among the shortlisted CpG sites, *FAR1* showed distinct hypermethylation in HCC tissues compared with adjacent non-tumour liver and other cancers (Figure 1(B)). *PAK1* also displayed elevated methylation in HCC, whereas *BDH1* exhibited a liver tissue-preferential pattern, with hypermethylation restricted to liver tissues across diverse cancer and normal samples (Supplemental Figure S1).

### Verification in cancer cell lines and primary tissues

Candidate markers were further evaluated in eight cancer cell lines, including two liver cancer cell lines (SNU-387 and SK-HEP-1) and six representing colon, stomach, pancreas, lung, breast, and prostate cancers. *FAR1* was consistently methylated in liver cancer cell lines and largely absent in other cancer cell types (Figure 1(C)). In contrast, *PAK1* yielded weak signals in SNU-387 and was also detected in a colon cancer cell line, indicating limited specificity. *BDH1* showed strong hypermethylation restricted to liver cancer cell lines, while remaining unmethylated in non-liver cancer cell types (Supplemental Figure S2A). Methylation levels were then assessed in 50 matched HCC tumour and non-tumour tissue pairs (Figure 1(D) and Supplemental Figure S2B). *FAR1* methylation was virtually absent in non-tumour tissues, underscoring its specificity. Although *PAK1* and *BDH1* were also significantly elevated in tumours, both showed occasional detection in non-tumour samples. Based on these findings, *FAR1* was prioritized as the lead candidate for subsequent plasma validation, although *PAK1* and *BDH1* were also further evaluated.

### Clinical validation of plasma DNA methylation markers for HCC detection

**Figure 2.**
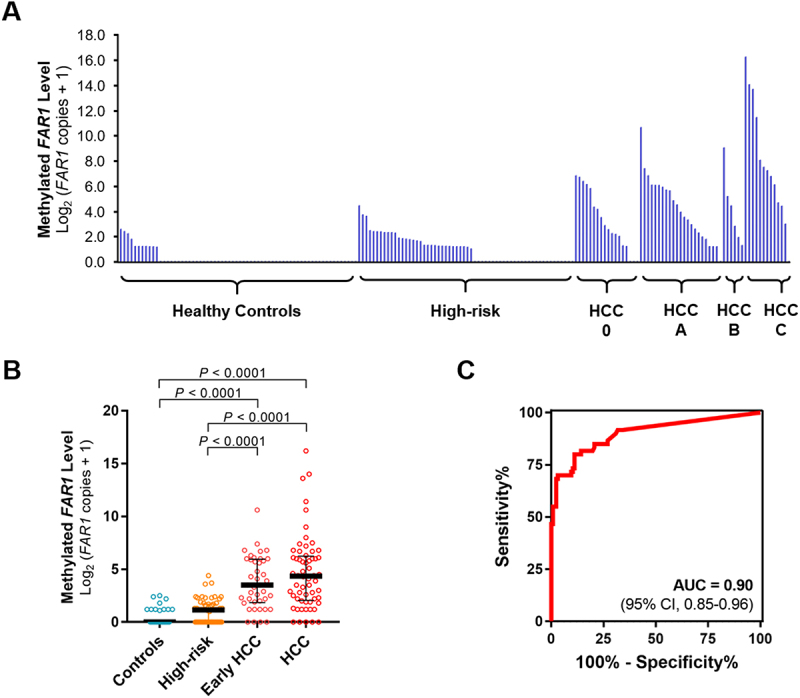
Detection of *FAR1* methylation status in plasma specimens of the validation cohort. (a, B) Digital PCR assay was performed to detect methylated *FAR1* in plasma samples from patients with hepatocellular carcinoma (HCC); high-risk individuals with hepatitis B virus, hepatitis C virus, alcoholic steatohepatitis, or metabolic dysfunction – associated steatotic liver disease; and healthy controls in the validation cohort. Early HCC included patients with stage 0–A classified according to BCLC (Barcelona Clinic Liver Cancer) staging system. Digital PCR results are presented as the median with interquartile range. Differences were assessed using one-way ANOVA followed by Tukey’s multiple comparison test. (C) Receiver-operating characteristic (ROC) curve for *FAR1* in discriminating HCC patients from non-HCC subjects in plasma specimens, presented with the AUC (area under the curve) value and 95% confidence intervals (CIs).

The assay was evaluated in a plasma cohort of 186 individuals (Table 1). To assess potential confounding by sex, a multivariable logistic regression model including plasma *FAR1* methylation and sex was performed, and *FAR1* remained independently associated with HCC status after adjustment for sex (Supplemental Table S5). Plasma methylation levels of *FAR1*, *PAK1*, and *BDH1* were all significantly higher in HCC patients compared with both healthy controls and CLD patients (Figure 2(A,B) and Supplemental Figure S3A). Among these, *FAR1* showed the clearest distinction across HCC and non-HCC groups, indicating high disease specificity, whereas *PAK1* and *BDH1* were elevated in CLD patients relative to healthy controls. All three markers showed stage-related increases in methylation levels, with *FAR1* significantly elevated in early-stage HCC (BCLC stage 0–A), supporting its potential for early detection. *PAK1* and *BDH1* followed similar trends. In ROC analysis, *FAR1* achieved the highest performance (AUC = 0.90; 95% CI, 0.85–0.96), outperforming *PAK1* (AUC = 0.84; 95% CI, 0.78–0.90) and *BDH1* (AUC = 0.76; 95% CI, 0.69–0.84) (Figure 2(C) and Supplemental Figure S3B).

### Diagnostic performance of biomarker combinations

**Figure 3.**
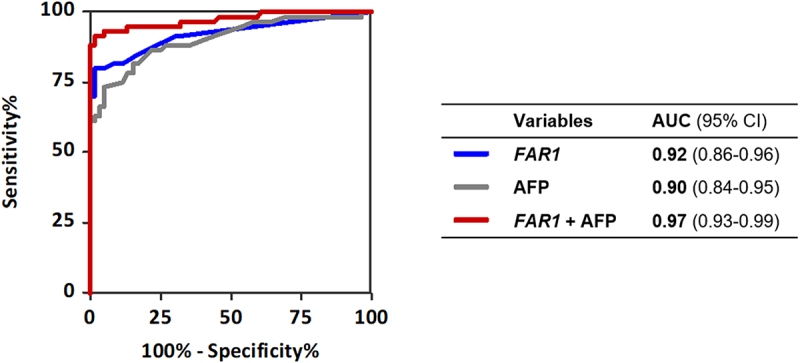
Diagnostic performance for *FAR1* methylation, serum alpha-fetoprotein (AFP), and their combination. AUC, area under the curve; CI, confidence interval.

At predefined cutoffs, *FAR1* alone achieved 70.0% sensitivity (95% CI, 56.8%–81.2%) and 96.8% specificity (95% CI, 92.1%–99.1%) (Table 2). A three-marker panel modestly improved sensitivity to 75.0% (95% CI, 62.1%–85.3%) with 93.7% specificity (95% CI, 87.9%–97.2%), exceeding the performance of serum AFP, which yielded 56.7% sensitivity (95% CI, 43.2%–69.4%) and 100.0% specificity (95% CI, 93.9%–100.0%) at a 12 ng/mL threshold (Supplemental Table S3 and Table 2). When combined with AFP, *FAR1* achieved 88.3% overall sensitivity (95% CI, 77.4%–95.2%) and 85.4% sensitivity in early-stage HCC (95% CI, 70.8%–94.4%), while maintaining 96.8% specificity. Logistic regression modelling further increased performance to 91.7% overall sensitivity (95% CI, 81.6%–97.2%), 90.2% early-stage sensitivity (95% CI, 76.9%–97.3%), and 98.3% specificity (95% CI, 90.9%–100.0%) (Supplemental Figure S4). The corresponding AUC was 0.97 (95% CI, 0.95–1.00) (Figure 3). Statistical comparison of ROC curves using DeLong’s test showed that the combination of *FAR1* and AFP resulted in a statistically significant improvement in AUC compared with AFP or *FAR1* alone (Supplemental Table S6). These findings support the *FAR1*-plus-AFP panel as a high-performance tool for HCC detection.

**Table 2.** Clinical performance of *FAR1* methylation in diagnosing hepatocellular carcinoma in the validation cohort.

Indicators (95% Confidence Interval)	*FAR1*	AFP (Cutoff: 12 ng/mL)	*FAR1* + AFP
Specificity	**96.8%** (92.1%-99.1%)	**100.0%** (93.9%-100.0%)	**96.8%** (92.1%-99.1%)
Overall Sensitivity	**70.0%** (56.8%-81.2%)	**56.7%** (43.2%-69.4%)	**88.3%** (77.4%-94.4%)
Early-stage Sensitivity (Stage 0-A)	**63.4%** (46.9%-77.9%)	**56.1%** (39.8%-71.5%)	**85.4%** (70.8%-94.4%)
Late-stage Sensitivity (Stage B-C)	**84.2%** (60.4%-96.6%)	**68.4%** (43.5%-87.4%)	**94.7%** (74.0%-99.9%)
Positive Predictive Value (PPV)	**91.3%** (79.2%-97.6%)	**100.0%** (89.7%-100.0%)	**93.0%** (83.0%-98.1%)
Negative Predictive Value (NPV)	**87.1%** (80.4%-92.2%)	**69.4%** (58.5%-79.0%)	**94.6%** (89.1%-97.8%)
Accuracy	**88.2%** (82.6%-92.4%)	**78.2%** (69.7%-85.2%)	**94.1%** (89.7%-97.0%)

### Association with clinicopathologic variables

Clinicopathologic correlation analysis revealed that *FAR1* methylation was not significantly associated with sex, age, cirrhosis, Child-Pugh class, or AFP levels (Table 3). *FAR1* methylation was significantly higher in advanced-stage HCC (BCLC stage B – C) than in early-stage disease (BCLC stage 0–A). Similar stage-related trends were observed for *PAK1* and *BDH1* (Supplemental Table S4). *FAR1* methylation significantly correlated with DCP levels but not with AFP, suggesting that AFP may provide complementary diagnostic value in combination with *FAR1*, whereas DCP may not. In contrast, *PAK1* and *BDH1* methylation levels were significantly associated with alanine transaminase (ALT). These findings suggest that *PAK1* and *BDH1* may primarily reflect underlying liver function and could serve as adjunctive markers for monitoring or treatment response, whereas *FAR1* remained tumour-specific and clinically relevant, highlighting its potential clinical utility in HCC surveillance.

**Table 3.** Correlation between clinicopathological variables and *FAR1* methylation in plasma samples.

Variables	Classification	*FAR1*^a^	*P*-value^b^
Sex	Male (*n* = 52)	4.7 ± 3.7	0.983
	Female (*n* = 8)	4.2 ± 2.0	
Age	≤60 (*n* = 32)	4.8 ± 4.5	0.830
	>60 (*n* = 28)	4.1 ± 2.7	
BCLC Stage	0-A (*n* = 41)	3.7 ± 2.5	**0.009**
	B-C (*n* = 19)	6.7 ± 4.5	
Cirrhosis	Yes (*n* = 49)	4.7 ± 3.6	0.767
	No (*n* = 11)	4.5 ± 3.0	
Child-Pugh Score	A (*n* = 50)	4.6 ± 3.3	0.620
	B-C (*n* = 8)	5.6 ± 4.9	
ALT	≤40 U/L (*n* = 44)	4.3 ± 3.4	0.098
	>40 U/L (*n* = 16)	5.8 ± 3.5	
AST	≤40 U/L (*n* = 36)	3.9 ± 2.5	0.072
	>40 U/L (*n* = 24)	6.0 ± 4.4	
AFP	<80 ng/mL (*n* = 24)	5.0 ± 3.5	0.330
	≥80 ng/mL (*n* = 36)	4.4 ± 3.5	
DCP	<40 mAu/mL (*n* = 21)	3.0 ± 2.3	**0.008**
	≥40 mAu/mL (*n* = 34)	5.6 ± 3.6	

^a^*FAR1* = Log_2_ (Methylated *FAR1* copies +1). Data were expressed as mean ± SD.

^b^Differences were evaluated by Mann-Whitney tests (*p* < 0.05was statistically significant and represented as bold).

BCLC, Barcelona Clinical Liver Cancer; ALT, alanine aminotransferase; AST, aspartate aminotransferase; AFP, alpha-fetoprotein; DCP, des-gamma-carboxy prothrombin.

## Discussion

A blood-based dPCR assay targeting DNA methylation markers for early HCC detection was developed and clinically validated. Three candidate biomarkers were identified through large-scale methylation analysis, among which *FAR1* demonstrated the strongest performance. In the overall cohort of 186 individuals, *FAR1* methylation achieved high diagnostic accuracy (AUC = 0.90). In the subset with available AFP data (*n* = 119), *FAR1* showed an AUC of 0.92 compared with 0.90 for AFP (Figure 3). Importantly, combining *FAR1* with AFP substantially improved diagnostic performance, yielding 88.3% overall sensitivity and 85.4% sensitivity in early-stage HCC while maintaining 96.8% specificity (AUC = 0.97) (Table 2 and Figure 3). These findings support the potential of *FAR1* methylation, particularly in combination with AFP, as a promising noninvasive biomarker for early detection and clinical surveillance of HCC.

While AFP remains widely used in clinical practice, its diagnostic utility is limited, particularly for early-stage HCC. Elevated AFP levels are frequently observed in non-malignant liver conditions such as chronic hepatitis or cirrhosis, whereas small or well-differentiated tumours often show low AFP expression [9,19]. Previous studies have reported AFP sensitivity ranging from 39% to 64%, depending on the threshold applied (10–20 ng/mL) [20,21]. To address these limitations, various combinatorial approaches have been investigated, including protein marker panels such as GALAD and methylation-based assays [11,12]. The GALAD score, which integrates AFP with additional serum markers and clinical variables, and plasma methylation markers such as *SEPT9* have demonstrated moderate diagnostic performance, with reported AUC values of approximately 0.75–0.85 across different cohorts. However, these approaches are either dependent on multiple clinical parameters or rely on single-marker assays, and their performance for early-stage HCC remains variable. Given these limitations, there is growing interest in combinatorial strategies that integrate epigenetic markers with AFP to further enhance early detection [13,14,22]. In this context, the *FAR1* methylation marker evaluated in this study demonstrated strong complementarity with AFP, resulting in improved diagnostic performance, particularly in early-stage HCC. These findings position *FAR1* methylation as a promising epigenetic component within AFP-based combinatorial frameworks, warranting further validation in prospective clinical studies.

The diagnostic relevance of the identified biomarkers may be supported by their biological roles in hepatocellular carcinogenesis. *FAR1* encodes a peroxisomal enzyme involved in lipid metabolism and ferroptosis regulation, and its depletion promotes tumour cell proliferation by inhibiting ether phospholipid synthesis [23]. Multi-omics analyses have shown reduced FAR1 mRNA expression in HCC despite stable protein levels, and *FAR1* was identified as one of 678 genes significantly hypermethylated in a genome-wide study of HCC tumours [24,25]. Although the functional consequences of this methylation remain unclear, *FAR1* methylation has been consistently associated with hepatocellular carcinogenesis. Similarly, *PAK1* is frequently overexpressed in HCC and contributes to tumour progression and metastasis through activation of epithelial-mesenchymal transition (EMT) pathways [26]. Specific promoter regions of *PAK1* have also been reported to be hypermethylated in HCC, showing positive correlations with mRNA expression and poor prognosis [27]. *BDH1*, a gene involved in ketone body metabolism, is downregulated in HCC and associated with adverse outcomes, potentially mediated by promoter methylation [28,29]. Collectively, these findings support the biological plausibility of these three methylation markers as HCC-associated biomarkers.

Beyond their role in distinguishing HCC from non-HCC cases, *PAK1* and *BDH1* may also serve as hepatocyte-derived cfDNA markers reflecting liver injury or dysfunction. In this study, both markers showed significantly higher methylation not only in HCC patients but also in individuals with CLD compared with healthy controls (Supplemental Figure S3), suggesting broader hepatic alterations including inflammation, fibrosis, or hepatocyte turnover. Previous studies have demonstrated the utility of hepatocyte-specific methylation markers for monitoring liver damage, transplant rejection, and treatment response [30,31]. For example, cfDNA methylation of genes such as *VTN* and *IGF2R* has been applied to quantify liver-derived cfDNA in transplantation and chemotherapy contexts [32]. Likewise, the observed correlation of *PAK1* and *BDH1* methylation with serum ALT levels supports their potential as indicators of liver injury (Supplemental Table S4). These findings suggest that, beyond HCC screening, *PAK1* and *BDH1* methylation may aid in assessing liver function and monitoring post-treatment response, including minimal residual disease detection and recurrence surveillance after resection or transplantation. Further validation in larger, longitudinal cohorts is warranted.

This study has several limitations. It was conducted in a single-centre, retrospective cohort that did not capture the full spectrum of high-risk patients, as most were Child-Pugh class A and cirrhosis patients without HCC were not included. In addition, the distribution of underlying liver disease etiologies was relatively limited, which may further restrict the generalizability of the findings. AFP values may also not fully reflect real-world practice due to limitations inherent in a retrospective design. Several features of the study help to mitigate these issues. Methylation levels were not significantly associated with cirrhosis status among HCC patients, suggesting limited impact of underlying cirrhosis on assay performance. Importantly, the consistent improvement observed when combining *FAR1* with AFP supports the reliability of the assay; however, future studies with refined cohorts that better reflect real-world practice will be required. Despite these limitations, this work demonstrates a promising blood-based assay that is comparable to – or exceeds – existing HCC biomarker panels, achieving higher sensitivity for early-stage HCC while maintaining high specificity. To fully establish its clinical utility, further large-scale, multicenter prospective studies including patients with diverse stages and underlying liver disease etiologies will be essential. With such validation, this dPCR platform could be integrated as a complementary blood-based tool within current HCC surveillance pathways for high-risk individuals undergoing routine imaging, and may further support posttreatment monitoring for early recurrence or minimal residual disease following curative-intent therapies, as well as post-transplant surveillance.

## Supplementary Material

Supplemental_Material_SJ_Park_et_al_v2R.docx

## Data Availability

DNA methylation profiles from TCGA were used and these datasets are publicly available at the Genomic Data Commons portal (https://portal.gdc.cancer.gov/). The summary-level data supporting the findings of this study are provided in the **Supplemental Material**. Additional detailed methylation data and raw dPCR data generated in this study are available from the corresponding author upon reasonable request for academic and non-commercial research purposes.
