# Supplementary material for: Development and validation of a digital PCR assay targeting plasma FAR1 methylation for early detection of hepatocellular carcinoma
Source: Epigenetics. 2026 Apr 20;21(1):2653956. doi: 10.1080/15592294.2026.2653956 (PMC13097777; doi:10.1080/15592294.2026.2653956)
Supplement: Supplemental_Material_SJ_Park_et_al_v2R.docx [file KEPI_A_2653956_SM4728.docx]

Supplemental Material

**Development and validation of a digital PCR assay targeting plasma *FAR1* methylation for early detection of hepatocellular carcinoma**

Sun Jae Park, Yun Young Lee, Young-Kyu Chung, Bo-Hyun Jung, Joon An, Jinil Han, Youngho Moon, and Hee-Jung Wang

TABLE OF CONTENTS

SUPPLeMENTal TABLES 2

SUPPLeMENTAl FIGURES 8

Raw and processed Data 12

DIGITAL MIQE CHECKLIST 18

**SUPPLEMENTAL TABLES**

**Supplemental Table S1.** Patient characteristics and demographics for tissue verification

| **Characteristics** | **Hepatocellular Carcinoma^a^** |
| --- | --- |
| **Total,** *n* | 50 |
| **Sex,** *n* **(%)** |  |
| Male | 32 (64.0) |
| Female | 18 (36.0) |
| **Age (years) - Median (range)** | 57 (35-86) |
| **mUICC Stage,** *n* **(%)** |  |
| I | 10 (20.0) |
| II | 13 (26.0) |
| III | 15 (30.0) |
| IV | 12 (24.0) |
| ^a^Tissue obtained from the Ajou University Hospital (Suwon, Republic of Korea)  mUICC, modified Union for International Cancer Control | |

**Supplemental Table S2.** Target regions of biomarker candidates and their biological roles

| **Biomarker** | **Biological Role** | **Genomic Coordinates^a^** | **CpG Sites in Amplicon^b^** | **Amplicon**  **Length (bp)** | |
| --- | --- | --- | --- | --- | --- |
| *FAR1*  (Fatty acyl-CoA reductase 1) | Peroxisome metabolism | Chr11:13689888-13690224 | Sense: 9  Antisense: 10 | Sense: 62  Antisense: 74 | |
| *PAK1*  (p21-activated kinase 1) | Cytoskeleton dynamics | Chr11:77122736-77123088 | Sense: 10 | Sense: 99 | |
| *BDH1*  (3-hydroxybutyrate dehydrogenase 1) | Ketone body metabolism | Chr3:197282603-197282949 | Sense: 10 | Sense: 57 | |
| ^a^GRCh37/hg19 assembly  ^b^Number of CpG sites encompassed within each digital PCR amplicon (strand-specific) | | | | |  |

**Supplemental Table S3.** Clinical performance of a plasma-based digital PCR assay using three biomarkers for detection of hepatocellular carcinoma in the validation cohort

| **Indicators** (95% CI) | **Three-marker Panel**^a^ | **Three-marker Panel + AFP** |
| --- | --- | --- |
| **Specificity** | **93.7%** (87.9%-97.2%) | **93.7%** (87.9%-97.2%) |
| **Overall Sensitivity** | **75.0%** (62.1%-85.3%) | **91.7%** (81.6%-97.2%) |
| **Early-stage Sensitivity** (Stage 0-A) | **65.9%** (49.4%-79.9%) | **87.8%** (73.8%-95.9%) |
| **Late-stage Sensitivity** (Stage B-C) | **89.5%** (66.9%-98.7%) | **94.7%** (74.0%-99.9%) |
| **PPV** | **84.9%** (72.4%-93.3%) | **87.3%** (76.5%-94.4%) |
| **NPV** | **88.7%** (82.1%-93.6%) | **95.9%** (90.8%-98.7%) |
| **Accuracy** | **87.6%** (82.0%-92.0%) | **93.0%** (88.3%-96.2%) |
| ^a^Three-marker Panel, A blood-based digital PCR assay using combination of three methylation biomarkers (*FAR1*, *PAK1*, and *BDH1*; named as HEPA eDX)  CI, confidence interval; AFP, alpha-fetoprotein; PPV, positive predictive value; NPV, negative predictive value | | |

**Supplemental Table S4.** Correlation between clinicopathological variables and methylation levels of *PAK1* or *BDH1* in plasma samples

| **Variables** | **Classification** | ***PAK1***^a^ | ***P*-value**^b^ | ***BDH1***^a^ | ***P*-value**^b^ |
| --- | --- | --- | --- | --- | --- |
| Sex | Male (*n* = 52) | 5.9 ± 2.4 | 0.572 | 7.0 ± 3.0 | 0.913 |
|  | Female (*n* = 8) | 5.4 ± 3.0 |  | 6.7 ± 2.7 |  |
| Age | ≤ 60 (*n* = 32) | 6.0 ± 3.0 | 0.744 | 7.2 ± 3.0 | 0.486 |
|  | > 60 (*n* = 28) | 5.8 ± 2.9 |  | 6.6 ± 2.9 |  |
| BCLC Stage | 0-A (*n* = 41) | 5.4 ± 2.8 | **0.044** | 6.4 ± 2.8 | 0.058 |
|  | B-C (*n* = 19) | 6.8 ± 3.0 |  | 8.0 ± 3.0 |  |
| Cirrhosis | Yes (*n* = 49) | 5.7 ± 2.9 | 0.172 | 6.7 ± 3.0 | 0.115 |
|  | No (*n* = 11) | 6.8 ± 2.8 |  | 8.0 ± 2.7 |  |
| Child-Pugh Class | A (*n* = 50) | 5.9 ± 2.9 | 0.857 | 6.9 ± 2.9 | 0.892 |
|  | B-C (*n* = 8) | 6.2 ± 3.4 |  | 7.4 ± 3.7 |  |
| ALT | ≤ 40 U/L (*n* = 44) | 5.6 ± 3.1 | **0.038** | 6.5 ± 3.1 | **0.015** |
|  | > 40 U/L (*n* = 16) | 6.7 ± 2.2 |  | 8.0 ± 2.0 |  |
| AST | ≤ 40 U/L (*n* = 36) | 5.4 ± 2.8 | 0.120 | 6.4 ± 2.9 | 0.091 |
|  | > 40 U/L (*n* = 24) | 6.5 ± 3.0 |  | 7.7 ± 3.0 |  |
| AFP | < 8 ng/mL (*n* = 24) | 6.3 ± 3.3 | 0.488 | 7.2 ± 3.4 | 0.667 |
|  | ≥ 8 ng/mL (*n* = 36) | 5.6 ± 2.6 |  | 6.7 ± 2.7 |  |
| DCP | < 40 mAu/mL (*n* = 21) | 5.4 ± 2.8 | 0.488 | 6.4 ± 2.8 | 0.391 |
|  | ≥ 40 mAu/mL (*n* = 34) | 6.1 ± 2.9 |  | 7.2 ± 3.1 |  |
| *FAR1*^a^ | Positive (*n* = 42) | 6.9 ± 2.7 | **< 0.0001** | 8.1 ± 2.7 | **< 0.0001** |
|  | Negative (*n* = 18) | 3.4 ± 1.3 |  | 4.3 ± 1.3 |  |
| ^a^Log_2_ (Target copies +1). Data were expressed as mean ± SD  ^b^Differences were evaluated by Mann-Whitney tests (*P* < 0.05 was statistically significant and represented as bold)  BCLC, Barcelona Clinical Liver Cancer; ALT, alanine aminotransferase; AST, aspartate aminotransferase; AFP, alpha-fetoprotein; DCP, des-gamma-carboxy prothrombin | | | | | |

**Supplemental Table S5.** Multivariable logistic regression models evaluating the association of plasma *FAR1* methylation with hepatocellular carcinoma status.

| **Model**^a^ | **Covariate** | ***N*** (HCC/Non-HCC)^b^ | **Odds Ratio** (95% CI) | ***P*-value** | **AUC** (95% CI) |
| --- | --- | --- | --- | --- | --- |
| 1 | *FAR1* | 186 (60/126) | 2.91 (2.06-4.08) | <0.0001 | 0.923  (0.874-0.957) |
|  | Sex |  | 0.26 (0.08-0.83) | 0.0229 |  |
| 2 | *FAR1* | 186 (60/126) | 2.78 (1.91-4.04) | <0.0001 | 0.948  (0.906-975) |
|  | Sex |  | 0.17 (0.05-0.61) | 0.0068 |  |
|  | Age |  | 1.10 (1.05-1.16) | 0.0002 |  |
| 3 | *FAR1* | 119 (60/59) | 3.64 (1.58-8.40) | 0.0025 | 0.985  (0.944-0.999) |
|  | Sex |  | 0.04 (0.00-1.36) | 0.0742 |  |
|  | Age |  | 1.08 (0.98-1.19) | 0.1039 |  |
|  | AFP |  | 1.43 (0.95-2.15) | 0.0852 |  |
| ^a^Model 1 includes *FAR1* methylation and sex. Model 2 includes *FAR1* methylation, sex, and age. Model 3 includes *FAR1* methylation, sex, age, and AFP.  ^b^Differences in sample size across models reflect the availability of AFP measurements for multivariate analysis.  AFP, alpha-fetoprotein; AUC, area under the receiver-operating characteristics curve; CI, confidence interval; HCC, hepatocellular carcinoma | | | | | |

**Supplemental Table S6.** Pairwise comparisons of diagnostic performance among AFP, *FAR1*, and their combination using DeLong’s test

| **Comparison** | **ΔAUC** (95% CI)^a^ | **DeLong *P*-value** | ***N***^b^ | |
| --- | --- | --- | --- | --- |
| AFP vs. *FAR1* | 0.015 (-0.056-0.086) | 0.676 | 119 | |
| AFP vs. *FAR1* + AFP combination | 0.069 (0.019-0.120) | 0.008 | 119 | |
| *FAR1* vs. *FAR1* + AFP combination | 0.054 (0.014-0.095) | 0.009 | 119 | |
| ^a^ΔAUC represents the difference in AUC between the two compared models.  ^b^All AUC comparisons were conducted using the same set of subjects with available AFP measurements, enabling paired comparisons of correlated ROC (receiver-operating characteristic) curves  AFP, alpha-fetoprotein; AUC, area under the receiver-operating characteristic curve; CI, confidence interval | | | |  |

**SUPPLEMENTAL FIGURES**

**Supplemental Figure S1.** DNA methylation levels of *PAK1* and *BDH1* across 33 cancer types and normal tissues (β-values represent the methylation level, ranging from 0 to 1). LIHC, liver hepatocellular carcinoma; CHOL, cholangiocarcinoma; LGG, Low grade glioma


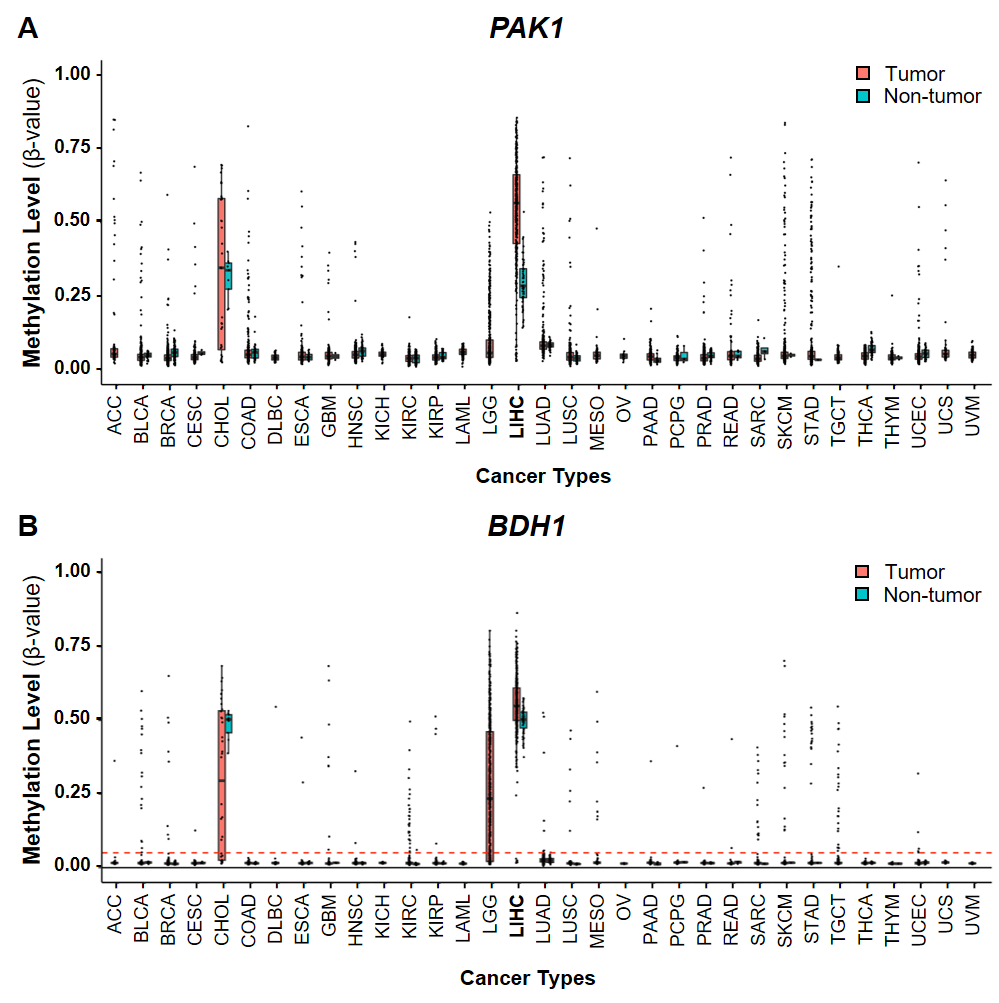


**Supplemental Figure S2.** DNA methylation status of *PAK1* and *BDH1* in cancer cell lines and liver cancer tissues. (**A**) DNA methylation levels of *PAK1* and *BDH1* in eight cancer cell lines, including two liver cancer cell lines (SNU-387 and SK-HEP-1) were analyzed. Cancer cell lines from colon (SW620), stomach (KATO-III), pancreas (ASPC-1), lung (HCC-827), breast (MCF7), and prostate (DU145) were also tested. Digital PCR results are presented as means ± SD from three independent experiments. Differences were assessed using one-way ANOVA followed by Tukey’s multiple comparison test (**P* < 0.0001). (**B**) DNA methylation levels of *PAK1* and *BDH1* were analyzed in primary tumors and paired adjacent non-tumor tissues from 50 hepatocellular carcinoma patients. Samples from the same patient are connected by a line. Differences were assessed using paired *t*-test.


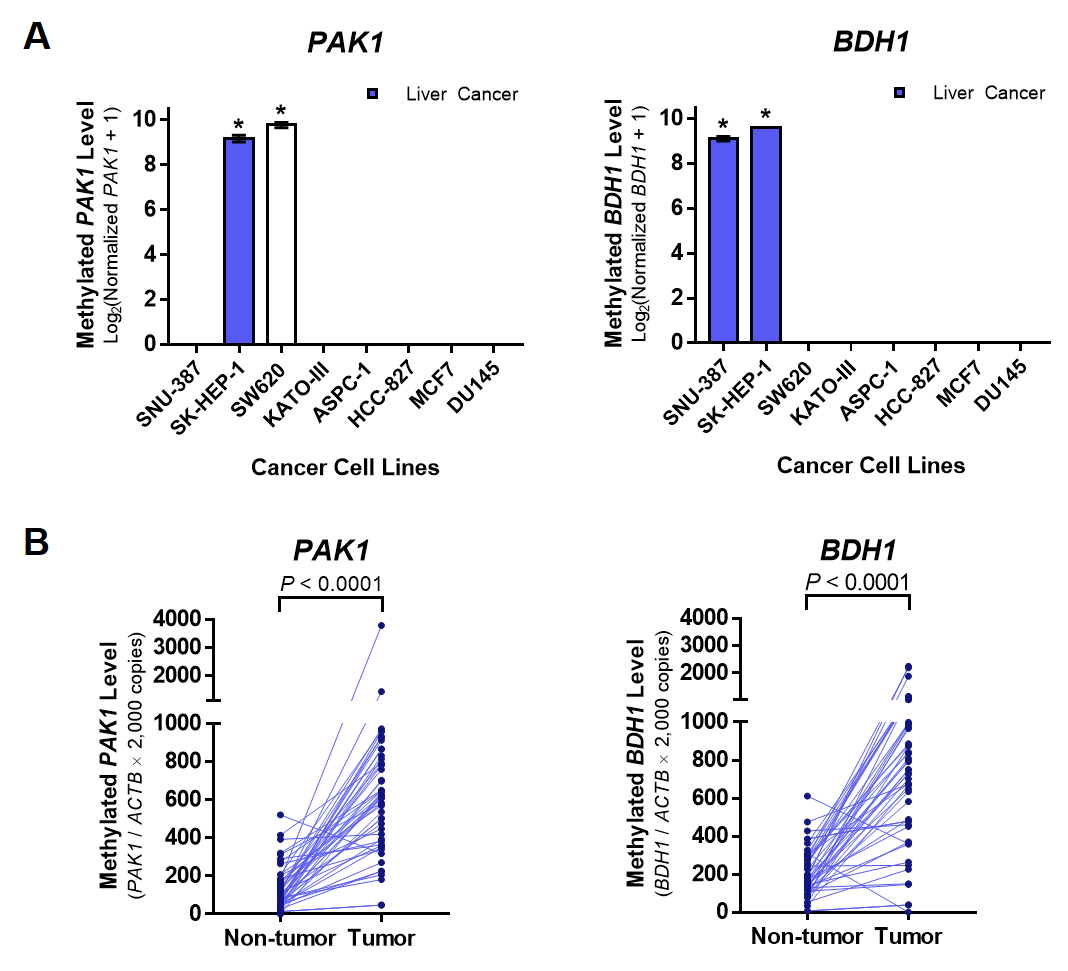


**Supplemental Figure S3.** Detection of *PAK1* and *BDH1* methylation status in plasma specimens from the validation cohort. (**A**) Digital PCR assay was performed to detect methylated *PAK1* and *BDH1* in plasma samples from HCC patients, high-risk patients, and healthy controls in the validation cohort. Early HCC (hepatocellular carcinoma) included patients with stage 0-A, classified according to Barcelona Clinic Liver Cancer (BCLC) staging system. Digital PCR results are presented as the median with interquartile range. Differences were assessed using one-way ANOVA followed by Tukey’s multiple comparison test. (**B**) Receiver-operating characteristic (ROC) curve for *PAK1* and *BDH1* as individual biomarkers for discriminating HCC patients from non-HCC subjects in plasma specimens, presented with AUC (area under the curve) values and 95% confidence intervals (CIs).


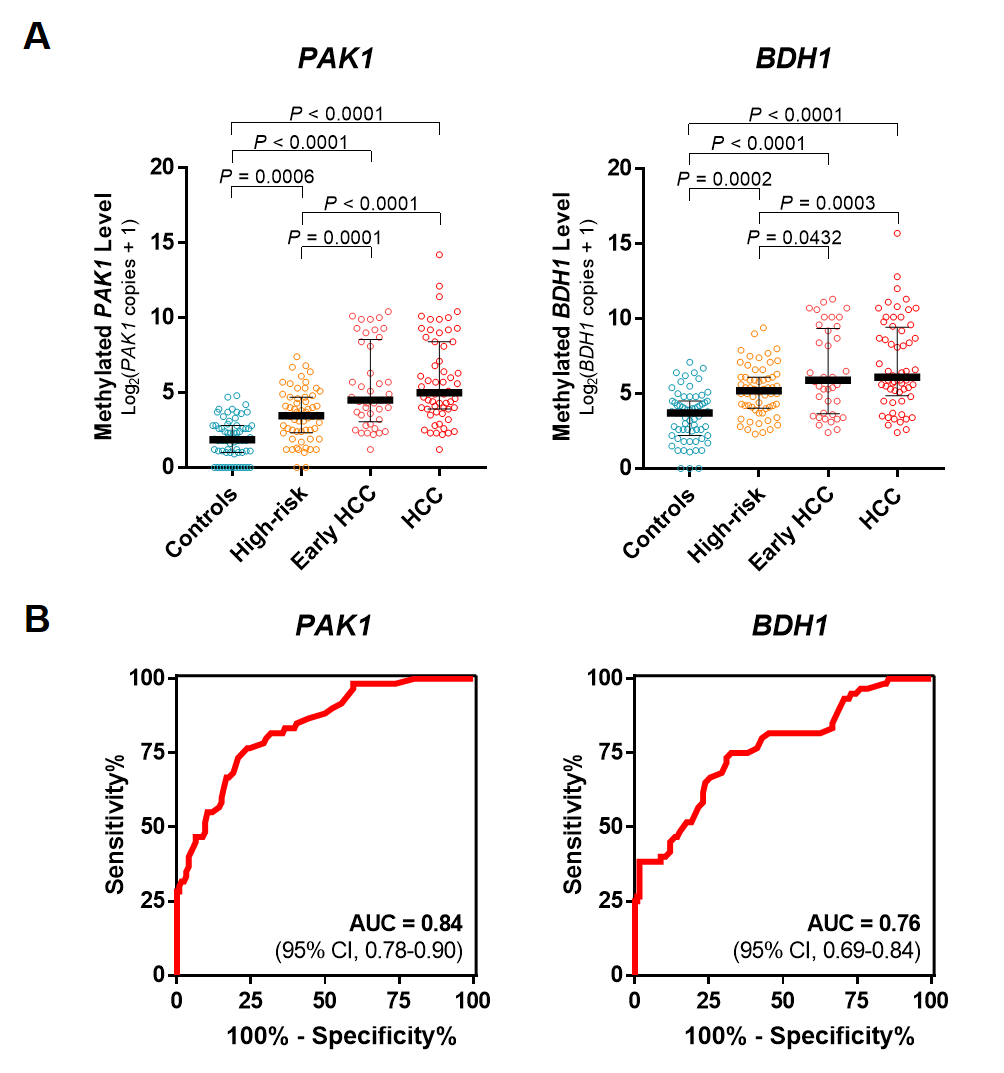


**Supplemental Figure S4.** Combination of methylated *FAR1* and AFP for hepatocellular carcinoma (HCC) detection using a logistic regression model. (**A**) Predicted probabilities for the combination of *FAR1* methylation AFP in each group, depicting using a box-and-whisker plot. The dotted line indicates values the threshold for 98% specificity. (**B**) Diagnostic performance of the logistic regression model for the combination of *FAR1* methylation and AFP. Error bars represent 95% Confidence intervals (CIs).

**
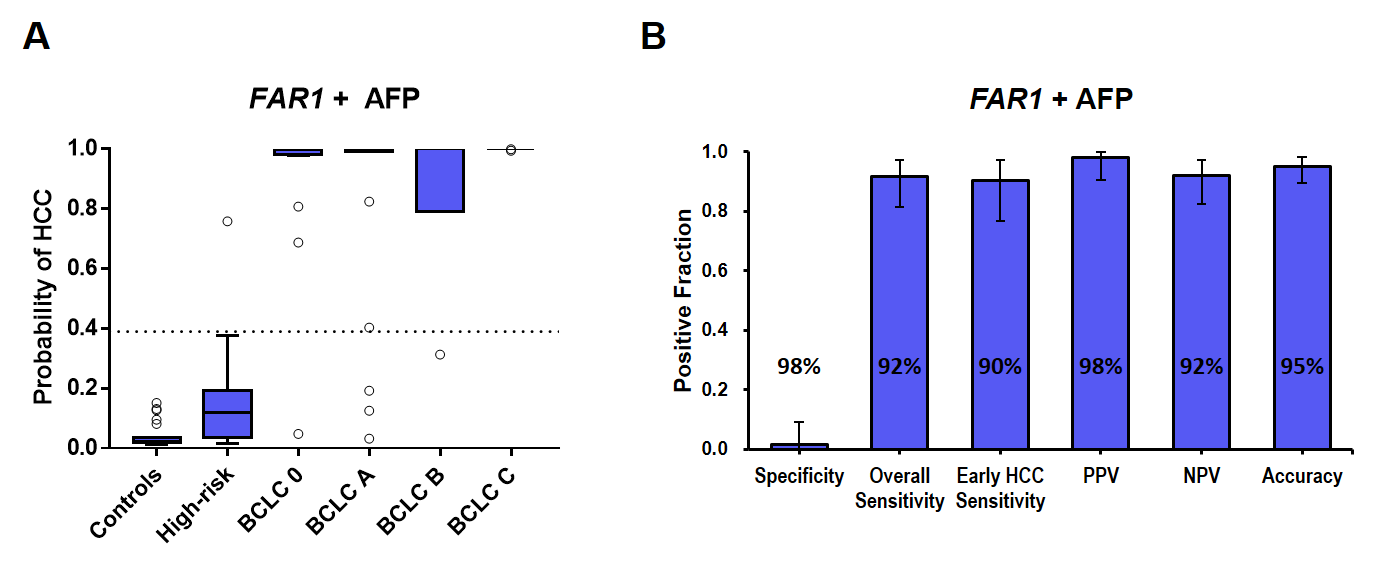
**

**RAW AND PROCESSED DATA**

| **Raw Data for Cancer Cell Line Tests:** | | | | | | | | | | | | | | | | | | | | | | | | | | | | | | | | |
| --- | --- | --- | --- | --- | --- | --- | --- | --- | --- | --- | --- | --- | --- | --- | --- | --- | --- | --- | --- | --- | --- | --- | --- | --- | --- | --- | --- | --- | --- | --- | --- | --- |
| Repeat | Target | | | | | NTC | | PC | | | SNU-387 | | | SK-HEP-1 | | | SW620 | | | KATO-III | | | ASPC-1 | | | HCC-827 | | | MCF7 | | | DU145 |
| Test 1 | FAR1 (copies) | | | | | 0.0 | | 878.0 | | | 376.6 | | | 366.9 | | | 0.0 | | | 0.0 | | | 0.0 | | | 0.0 | | | 0.0 | | | 0.0 |
|  | PAK1 (copies) | | | | | 0.0 | | 303.5 | | | 0.0 | | | 341.6 | | | 442.9 | | | 0.0 | | | 0.0 | | | 0.0 | | | 0.0 | | | 0.0 |
|  | BDH1 (copies) | | | | | 0.0 | | 455.3 | | | 197.8 | | | 416.4 | | | 0.0 | | | 0.0 | | | 0.0 | | | 0.0 | | | 0.0 | | | 0.0 |
|  | ACTB (copies) | | | | | 0.0 | | 662.6 | | | 702.6 | | | 1076.9 | | | 1032.0 | | | 1822.2 | | | 915.1 | | | 741.0 | | | 903.8 | | | 854.0 |
|  | Accepted Droplets | | | | | 19337 | | 20350 | | | 19789 | | | 20166 | | | 19482 | | | 19221 | | | 18953 | | | 18570 | | | 19950 | | | 21231 |
| Test 2 | FAR1 (copies) | | | | | 0.0 | | 947.6 | | | 379.7 | | | 378.0 | | | 0.0 | | | 0.0 | | | 0.0 | | | 0.0 | | | 0.0 | | | 0.0 |
|  | PAK1 (copies) | | | | | 0.0 | | 261.8 | | | 0.0 | | | 305.7 | | | 397.9 | | | 0.0 | | | 0.0 | | | 0.0 | | | 0.0 | | | 0.0 |
|  | BDH1 (copies) | | | | | 0.0 | | 453.0 | | | 214.6 | | | 413.6 | | | 0.0 | | | 0.0 | | | 0.0 | | | 0.0 | | | 0.0 | | | 0.0 |
|  | ACTB (copies) | | | | | 0.0 | | 645.7 | | | 710.5 | | | 1045.7 | | | 945.5 | | | 1858.6 | | | 809.9 | | | 674.7 | | | 957.3 | | | 1249.8 |
|  | Accepted Droplets | | | | | 20173 | | 19612 | | | 19427 | | | 20116 | | | 19818 | | | 18591 | | | 20960 | | | 20105 | | | 20408 | | | 20221 |
| Test 3 | FAR1 (copies) | | | | | 0.0 | | 87.61 | | | 355.3 | | | 346.4 | | | 0.0 | | | 0.0 | | | 0.0 | | | 0.0 | | | 0.0 | | | 0.0 |
|  | PAK1 (copies) | | | | | 0.0 | | 302.3 | | | 0.0 | | | 260.7 | | | 418.0 | | | 0.0 | | | 0.0 | | | 0.0 | | | 0.0 | | | 0.0 |
|  | BDH1 (copies) | | | | | 0.0 | | 427.1 | | | 155.4 | | | 398.3 | | | 0.0 | | | 0.0 | | | 0.0 | | | 0.0 | | | 0.0 | | | 0.0 |
|  | ACTB (copies) | | | | | 0.0 | | 629.2 | | | 602.2 | | | 1003.5 | | | 865.2 | | | 1715.2 | | | 880.8 | | | 662.2 | | | 887.6 | | | 1184.2 |
|  | Accepted Droplets | | | | | 19912 | | 19839 | | | 20463 | | | 18719 | | | 21239 | | | 20756 | | | 20230 | | | 20708 | | | 19789 | | | 19400 |
| NTC, no template control; PC, positive control | | | | | | | | | | | | | | | | | | | | | | | | | | | | | | | | |
|  | | | | | | | | | | | | | | | | | | | | | | | | | | | | | | | | |
| **Raw Data for Tissue Test:** | | | | | | | | | | | | | | | | | | | | | | | | | | | | | | | | |
| Sample ID | | | Sex | Age | | mUICC Stage | | | Adjacent non-tumor | | | | | | | | | | | | | Tumor | | | | | | | | | | |
|  |  |  |  |  |  |  |  |  | FAR1 (copies) | | | PAK1 (copies) | | | | BDH1 (copies) | | | ACTB (copies) | | | FAR1 (copies) | | | PAK1 (copies) | | | BDH1 (copies) | | | ACTB (copies) | |
| 82898 | | | F | 74 | | I | | | 1.0 | | | 98.0 | | | | 40.0 | | | 292.0 | | | 45.0 | | | 0.0 | | | 292.0 | | | 904.0 | |
| 674894 | | | F | 53 | | I | | | 7.0 | | | 487.0 | | | | 18.0 | | | 182.0 | | | 17.0 | | | 211.0 | | | 440.0 | | | 399.0 | |
| 150864 | | | F | 60 | | I | | | 0.0 | | | 0.0 | | | | 9.0 | | | 296.0 | | | 16.0 | | | 185.0 | | | 279.0 | | | 635.0 | |
| 7026204 | | | F | 78 | | I | | | 0.0 | | | 406.0 | | | | 7.0 | | | 306.0 | | | 58.0 | | | 383.0 | | | 401.0 | | | 871.0 | |
| 182343 | | | F | 66 | | I | | | 0.0 | | | 354.0 | | | | 0.0 | | | 166.0 | | | 15.0 | | | 227.0 | | | 581.0 | | | 519.0 | |
| 481170 | | | F | 59 | | I | | | 2.0 | | | 0.0 | | | | 9.0 | | | 289.0 | | | 28.0 | | | 342.0 | | | 665.0 | | | 1077.0 | |
| 325925 | | | F | 54 | | I | | | 9.0 | | | 399.0 | | | | 11.0 | | | 85.0 | | | 21.0 | | | 85.0 | | | 798.0 | | | 754.0 | |
| 399295 | | | F | 79 | | I | | | 1.0 | | | 60.0 | | | | 83.0 | | | 154.0 | | | 111.0 | | | 109.0 | | | 519.0 | | | 473.0 | |
| 779494 | | | F | 58 | | I | | | 4.0 | | | 106.0 | | | | 28.0 | | | 106.0 | | | 63.0 | | | 129.0 | | | 918.0 | | | 344.0 | |
| 568811 | | | F | 81 | | I | | | 0.0 | | | 428.0 | | | | 14.0 | | | 92.0 | | | 52.0 | | | 493.0 | | | 556.0 | | | 524.0 | |
| 25068276 | | | M | 50 | | II | | | 6.0 | | | 106.0 | | | | 94.0 | | | 130.0 | | | 99.0 | | | 356.0 | | | 712.0 | | | 677.0 | |
| 25199169 | | | M | 76 | | II | | | 0.0 | | | 8.0 | | | | 2.0 | | | 15.0 | | | 1.0 | | | 12.0 | | | 289.0 | | | 628.0 | |
| 25734828 | | | M | 61 | | II | | | 2.0 | | | 191.0 | | | | 8.0 | | | 87.0 | | | 52.0 | | | 83.0 | | | 269.0 | | | 346.0 | |
| 25233475 | | | M | 48 | | II | | | 2.0 | | | 0.0 | | | | 34.0 | | | 125.0 | | | 36.0 | | | 159.0 | | | 573.0 | | | 329.0 | |
| 25114596 | | | M | 78 | | II | | | 6.0 | | | 14.0 | | | | 123.0 | | | 370.0 | | | 62.0 | | | 698.0 | | | 626.0 | | | 1762.0 | |
| 25468218 | | | M | 62 | | II | | | 1.0 | | | 187.0 | | | | 12.0 | | | 168.0 | | | 27.0 | | | 73.0 | | | 374.0 | | | 552.0 | |
| 25295551 | | | M | 62 | | II | | | 8.0 | | | 1327.0 | | | | 60.0 | | | 976.0 | | | 130.0 | | | 890.0 | | | 945.0 | | | 2007.0 | |
| 25153184 | | | M | 61 | | II | | | 1.0 | | | 140.0 | | | | 7.0 | | | 164.0 | | | 31.0 | | | 161.0 | | | 383.0 | | | 403.0 | |
| 25182215 | | | F | 64 | | II | | | 1.0 | | | 590.0 | | | | 14.0 | | | 404.0 | | | 43.0 | | | 486.0 | | | 336.0 | | | 932.0 | |
| 25500027 | | | M | 55 | | II | | | 2.0 | | | 14.0 | | | | 39.0 | | | 32.0 | | | 56.0 | | | 89.0 | | | 674.0 | | | 178.0 | |
| 25674197 | | | F | 45 | | II | | | 0.0 | | | 1272.0 | | | | 54.0 | | | 460.0 | | | 85.0 | | | 548.0 | | | 520.0 | | | 956.0 | |
| 25320557 | | | M | 51 | | II | | | 6.0 | | | 268.0 | | | | 29.0 | | | 230.0 | | | 46.0 | | | 565.0 | | | 313.0 | | | 1355.0 | |
| 25395357 | | | M | 56 | | II | | | 3.0 | | | 983.0 | | | | 12.0 | | | 406.0 | | | 9.0 | | | 327.0 | | | 549.0 | | | 977.0 | |
| 25003089 | | | M | 86 | | III | | | 0.0 | | | 187.0 | | | | 11.0 | | | 510.0 | | | 41.0 | | | 585.0 | | | 318.0 | | | 1177.0 | |
| 25675441 | | | M | 70 | | III | | | 0.0 | | | 8.0 | | | | 2.0 | | | 15.0 | | | 1.0 | | | 12.0 | | | 289.0 | | | 628.0 | |
| 25422654 | | | M | 42 | | III | | | 2.0 | | | 191.0 | | | | 8.0 | | | 87.0 | | | 52.0 | | | 83.0 | | | 269.0 | | | 346.0 | |
| 25459002 | | | M | 55 | | III | | | 55.0 | | | 553.0 | | | | 64.0 | | | 224.0 | | | 74.0 | | | 393.0 | | | 407.0 | | | 770.0 | |
| 25500997 | | | M | 53 | | III | | | 3.0 | | | 3.0 | | | | 16.0 | | | 94.0 | | | 28.0 | | | 119.0 | | | 453.0 | | | 240.0 | |
| 25102279 | | | M | 52 | | III | | | 1.0 | | | 267.0 | | | | 11.0 | | | 95.0 | | | 33.0 | | | 205.0 | | | 217.0 | | | 400.0 | |
| 25212670 | | | M | 57 | | III | | | 5.0 | | | 38.0 | | | | 7.0 | | | 144.0 | | | 11.0 | | | 147.0 | | | 244.0 | | | 301.0 | |
| 25694909 | | | M | 57 | | III | | | 0.0 | | | 787.0 | | | | 13.0 | | | 817.0 | | | 13.0 | | | 483.0 | | | 142.0 | | | 431.0 | |
| 25584230 | | | M | 54 | | III | | | 3.0 | | | 4.0 | | | | 26.0 | | | 81.0 | | | 31.0 | | | 101.0 | | | 342.0 | | | 278.0 | |
| 25659156 | | | M | 57 | | III | | | 0.0 | | | 354.0 | | | | 12.0 | | | 186.0 | | | 12.0 | | | 187.0 | | | 276.0 | | | 533.0 | |
| 25586976 | | | F | 55 | | III | | | 1.0 | | | 1038.0 | | | | 28.0 | | | 361.0 | | | 36.0 | | | 315.0 | | | 437.0 | | | 779.0 | |
| 25117156 | | | M | 40 | | III | | | 1.0 | | | 156.0 | | | | 32.0 | | | 133.0 | | | 45.0 | | | 173.0 | | | 395.0 | | | 411.0 | |
| 25735529 | | | F | 66 | | III | | | 0.0 | | | 234.0 | | | | 17.0 | | | 624.0 | | | 27.0 | | | 195.0 | | | 310.0 | | | 865.0 | |
| 25350579 | | | F | 55 | | III | | | 6.0 | | | 343.0 | | | | 128.0 | | | 135.0 | | | 79.0 | | | 195.0 | | | 618.0 | | | 341.0 | |
| 25152731 | | | M | 48 | | III | | | 5.0 | | | 0.0 | | | | 56.0 | | | 218.0 | | | 78.0 | | | 1067.0 | | | 546.0 | | | 975.0 | |
| 25424684 | | | F | 35 | | IV | | | 0.0 | | | 579.0 | | | | 54.0 | | | 194.0 | | | 122.0 | | | 229.0 | | | 514.0 | | | 681.0 | |
| 25060022 | | | M | 37 | | IV | | | 6.0 | | | 291.0 | | | | 128.0 | | | 79.0 | | | 150.0 | | | 90.0 | | | 491.0 | | | 498.0 | |
| 25424891 | | | M | 58 | | IV | | | 5.0 | | | 249.0 | | | | 73.0 | | | 106.0 | | | 76.0 | | | 197.0 | | | 503.0 | | | 582.0 | |
| 25749229 | | | M | 49 | | IV | | | 0.0 | | | 187.0 | | | | 19.0 | | | 119.0 | | | 29.0 | | | 98.0 | | | 449.0 | | | 1307.0 | |
| 25244983 | | | F | 64 | | IV | | | 6.0 | | | 537.0 | | | | 22.0 | | | 214.0 | | | 12.0 | | | 127.0 | | | 251.0 | | | 686.0 | |
| 25455213 | | | M | 57 | | IV | | | 0.0 | | | 894.0 | | | | 29.0 | | | 312.0 | | | 27.0 | | | 257.0 | | | 351.0 | | | 791.0 | |
| 25003063 | | | M | 46 | | IV | | | 1.0 | | | 292.0 | | | | 9.0 | | | 96.0 | | | 31.0 | | | 173.0 | | | 405.0 | | | 709.0 | |
| 25501522 | | | M | 57 | | IV | | | 0.0 | | | 283.0 | | | | 9.0 | | | 42.0 | | | 19.0 | | | 140.0 | | | 338.0 | | | 373.0 | |
| 25555176 | | | M | 57 | | IV | | | 0.0 | | | 20.0 | | | | 38.0 | | | 141.0 | | | 67.0 | | | 57.0 | | | 550.0 | | | 461.0 | |
| 25579447 | | | M | 50 | | IV | | | 0.0 | | | 0.0 | | | | 16.0 | | | 209.0 | | | 43.0 | | | 389.0 | | | 640.0 | | | 1062.0 | |
| 25645474 | | | F | 54 | | IV | | | 1.0 | | | 0.0 | | | | 13.0 | | | 96.0 | | | 17.0 | | | 68.0 | | | 306.0 | | | 932.0 | |
| 25720246 | | | M | 60 | | IV | | | 6.0 | | | 414.0 | | | | 34.0 | | | 246.0 | | | 43.0 | | | 142.0 | | | 639.0 | | | 792.0 | |
| mUICC, modified Union for International Cancer Control | | | | | | | | | | | | | | | | | | | | | | | | | | | | | | | | |
|  | | | | | | | | | | | | | | | | | | | | | | | | | | | | | | | | |
| **Raw Data for Plasma Test:** | | | | | | | | | | | | | | | | | | | | | | | | | | | | | | | | |
| Sample ID | | Group | | | Disease | | BCLC Stage | | | Sex | | | Age | | AFP (ng/mL) | | | FAR1 (copies) | | | PAK1 (copies) | | | BDH1 (copies) | | | ACTB (copies) | | | Accepted Droplet | | |
| 1210153 | | Controls | | | None | | None | | | F | | | 54 | | n/a | | | 1.3 | | | 0.0 | | | 6.4 | | | 5971.0 | | | 19572 | | |
| 1210154 | | Controls | | | None | | None | | | F | | | 57 | | n/a | | | 0.0 | | | 4.0 | | | 5.0 | | | 2001.0 | | | 19880 | | |
| 1210155 | | Controls | | | None | | None | | | F | | | 57 | | n/a | | | 0.0 | | | 1.0 | | | 17.0 | | | 4964.0 | | | 19472 | | |
| 1210158 | | Controls | | | None | | None | | | F | | | 56 | | n/a | | | 3.6 | | | 17.0 | | | 85.0 | | | 4157.0 | | | 20762 | | |
| 1210159 | | Controls | | | None | | None | | | F | | | 41 | | n/a | | | 0.0 | | | 1.0 | | | 5.0 | | | 6028.0 | | | 21532 | | |
| 1210161 | | Controls | | | None | | None | | | F | | | 35 | | n/a | | | 0.0 | | | 3.0 | | | 15.0 | | | 9999.0 | | | 18014 | | |
| 1210164 | | Controls | | | None | | None | | | F | | | 41 | | n/a | | | 0.0 | | | 4.9 | | | 3.7 | | | 14389.0 | | | 20448 | | |
| 1210167 | | Controls | | | None | | None | | | F | | | 26 | | n/a | | | 0.0 | | | 1.2 | | | 2.5 | | | 5588.0 | | | 20503 | | |
| 1210168 | | Controls | | | None | | None | | | F | | | 23 | | n/a | | | 0.0 | | | 0.0 | | | 1.2 | | | 2310.0 | | | 20376 | | |
| 46832 | | Controls | | | None | | None | | | F | | | 62 | | n/a | | | 0.0 | | | 12.0 | | | 15.0 | | | 340.0 | | | 20215 | | |
| 46479 | | Controls | | | None | | None | | | M | | | 39 | | n/a | | | 0.0 | | | 6.2 | | | 18.5 | | | 1855.0 | | | 20453 | | |
| 46046 | | Controls | | | None | | None | | | M | | | 36 | | n/a | | | 4.2 | | | 25.3 | | | 133.6 | | | 9860.0 | | | 17936 | | |
| 46833 | | Controls | | | None | | None | | | F | | | 40 | | n/a | | | 0.0 | | | 0.0 | | | 16.0 | | | 2219.0 | | | 19153 | | |
| 46830 | | Controls | | | None | | None | | | M | | | 63 | | n/a | | | 0.0 | | | 3.0 | | | 5.0 | | | 1826.0 | | | 19757 | | |
| 46873 | | Controls | | | None | | None | | | F | | | 37 | | n/a | | | 1.2 | | | 11.8 | | | 30.7 | | | 933.0 | | | 21338 | | |
| 46471 | | Controls | | | None | | None | | | F | | | 24 | | n/a | | | 0.0 | | | 0.0 | | | 1.3 | | | 19333.8 | | | 20006 | | |
| 46475 | | Controls | | | None | | None | | | F | | | 69 | | n/a | | | 0.0 | | | 2.0 | | | 0.0 | | | 2724.0 | | | 20310 | | |
| 1135906 | | Controls | | | None | | None | | | F | | | 45 | | n/a | | | 0.0 | | | 1.0 | | | 22.0 | | | 895.0 | | | 21371 | | |
| 46630 | | Controls | | | None | | None | | | F | | | 69 | | n/a | | | 0.0 | | | 1.3 | | | 2.5 | | | 3390.0 | | | 19996 | | |
| 46477 | | Controls | | | None | | None | | | F | | | 21 | | n/a | | | 0.0 | | | 1.0 | | | 3.0 | | | 2172.0 | | | 19597 | | |
| 1210163 | | Controls | | | None | | None | | | F | | | 55 | | n/a | | | 0.0 | | | 1.2 | | | 26.4 | | | 2533.4 | | | 20982 | | |
| 1210165 | | Controls | | | None | | None | | | F | | | 73 | | n/a | | | 0.0 | | | 14.5 | | | 43.4 | | | 2495.5 | | | 19156 | | |
| 1210169 | | Controls | | | None | | None | | | F | | | 54 | | n/a | | | 0.0 | | | 1.2 | | | 12.1 | | | 6436.1 | | | 20879 | | |
| 1210170 | | Controls | | | None | | None | | | F | | | 54 | | n/a | | | 0.0 | | | 2.4 | | | 6.0 | | | 2555.3 | | | 20987 | | |
| 1210171 | | Controls | | | None | | None | | | F | | | 51 | | n/a | | | 0.0 | | | 4.9 | | | 29.3 | | | 4130.5 | | | 20645 | | |
| 43834 | | Controls | | | None | | None | | | F | | | 25 | | n/a | | | 0.0 | | | 0.0 | | | 13.5 | | | 3260.8 | | | 20507 | | |
| 46868 | | Controls | | | None | | None | | | M | | | 51 | | n/a | | | 1.3 | | | 3.9 | | | 13.0 | | | 2826.6 | | | 20318 | | |
| 46045 | | Controls | | | None | | None | | | M | | | 27 | | n/a | | | 1.3 | | | 6.5 | | | 15.6 | | | 6487.9 | | | 20318 | | |
| 46398 | | Controls | | | None | | None | | | F | | | 25 | | n/a | | | 0.0 | | | 0.0 | | | 6.1 | | | 4594.0 | | | 20580 | | |
| 46875 | | Controls | | | None | | None | | | F | | | 65 | | n/a | | | 0.0 | | | 9.1 | | | 5.2 | | | 5755.0 | | | 19454 | | |
| 46469 | | Controls | | | None | | None | | | F | | | 33 | | n/a | | | 0.0 | | | 0.0 | | | 2.9 | | | 3621.3 | | | 20275 | | |
| 46472 | | Controls | | | None | | None | | | M | | | 35 | | n/a | | | 0.0 | | | 3.0 | | | 12.1 | | | 1137.7 | | | 19597 | | |
| 46473 | | Controls | | | None | | None | | | M | | | 35 | | n/a | | | 0.0 | | | 2.5 | | | 2.5 | | | 8433.5 | | | 20465 | | |
| 46474 | | Controls | | | None | | None | | | M | | | 35 | | n/a | | | 0.0 | | | 4.3 | | | 20.3 | | | 9932.7 | | | 19300 | | |
| 46476 | | Controls | | | None | | None | | | F | | | 41 | | n/a | | | 0.0 | | | 0.0 | | | 2.4 | | | 3292.5 | | | 21333 | | |
| 4230909 | | Controls | | | None | | None | | | M | | | 23 | | n/a | | | 0.0 | | | 2.4 | | | 32.3 | | | 1406.9 | | | 21015 | | |
| 4230927 | | Controls | | | None | | None | | | M | | | 56 | | n/a | | | 0.0 | | | 5.0 | | | 17.5 | | | 7988.0 | | | 20136 | | |
| 0401 | | Controls | | | None | | None | | | M | | | 49 | | n/a | | | 4.8 | | | 2.4 | | | 19.4 | | | 2654.5 | | | 20751 | | |
| 4771409 | | Controls | | | None | | None | | | M | | | 36 | | n/a | | | 0.0 | | | 4.9 | | | 9.9 | | | 13719.9 | | | 20413 | | |
| 4771410 | | Controls | | | None | | None | | | M | | | 25 | | n/a | | | 0.0 | | | 6.1 | | | 1.2 | | | 4857.5 | | | 20531 | | |
| 220037 | | Controls | | | None | | None | | | F | | | 47 | | 1.5 | | | 0.0 | | | 7.5 | | | 13.8 | | | 2606.2 | | | 20130 | | |
| 220126 | | Controls | | | None | | None | | | F | | | 45 | | 1.7 | | | 1.3 | | | 1.3 | | | 26.6 | | | 2165.2 | | | 19875 | | |
| 220184 | | Controls | | | None | | None | | | F | | | 44 | | 1.1 | | | 0.0 | | | 0.0 | | | 1.3 | | | 1750.8 | | | 19471 | | |
| 220190 | | Controls | | | None | | None | | | M | | | 52 | | 1.8 | | | 0.0 | | | 3.8 | | | 15.1 | | | 1149.3 | | | 20019 | | |
| 220239 | | Controls | | | None | | None | | | F | | | 44 | | 2.3 | | | 0.0 | | | 5.0 | | | 60.8 | | | 2277.1 | | | 20291 | | |
| 220278 | | Controls | | | None | | None | | | F | | | 50 | | 2.7 | | | 1.3 | | | 2.5 | | | 8.8 | | | 963.5 | | | 20094 | | |
| 230013 | | Controls | | | None | | None | | | F | | | 49 | | 1.6 | | | 0.0 | | | 0.0 | | | 10.1 | | | 1377.4 | | | 27422 | | |
| 230349 | | Controls | | | None | | None | | | F | | | 45 | | 1.2 | | | 0.0 | | | 0.8 | | | 8.4 | | | 926.0 | | | 29941 | | |
| 230406 | | Controls | | | None | | None | | | F | | | 49 | | 0.5 | | | 0.0 | | | 0.0 | | | 0.0 | | | 974.1 | | | 24644 | | |
| 240009 | | Controls | | | None | | None | | | F | | | 51 | | 1.3 | | | 0.0 | | | 2.6 | | | 1.8 | | | 1199.0 | | | 28705 | | |
| 240030 | | Controls | | | None | | None | | | F | | | 46 | | 1.3 | | | 0.0 | | | 12.1 | | | 74.9 | | | 7981.0 | | | 20850 | | |
| 240122 | | Controls | | | None | | None | | | M | | | 52 | | 1.9 | | | 0.0 | | | 1.2 | | | 17.0 | | | 2266.6 | | | 20776 | | |
| 240246 | | Controls | | | None | | None | | | F | | | 50 | | 1.5 | | | 0.0 | | | 0.0 | | | 0.0 | | | 1362.5 | | | 21283 | | |
| 240362 | | Controls | | | None | | None | | | F | | | 45 | | 1.6 | | | 0.0 | | | 1.2 | | | 1.2 | | | 743.6 | | | 20224 | | |
| 240378 | | Controls | | | None | | None | | | F | | | 50 | | 1.1 | | | 0.0 | | | 6.1 | | | 3.7 | | | 2089.3 | | | 20503 | | |
| 240427 | | Controls | | | None | | None | | | F | | | 50 | | 0.9 | | | 0.0 | | | 3.9 | | | 26.0 | | | 2685.0 | | | 19342 | | |
| 230003 | | Controls | | | None | | None | | | M | | | 46 | | 1.8 | | | 0.0 | | | 12.2 | | | 41.5 | | | 2562.0 | | | 20611 | | |
| 230355 | | Controls | | | None | | None | | | M | | | 50 | | 1.6 | | | 0.0 | | | 10.8 | | | 21.6 | | | 1518.0 | | | 21005 | | |
| 240018 | | Controls | | | None | | None | | | M | | | 51 | | 0.9 | | | 0.0 | | | 0.0 | | | 7.3 | | | 1542.1 | | | 21729 | | |
| 240123 | | Controls | | | None | | None | | | M | | | 52 | | 1.0 | | | 2.4 | | | 27.6 | | | 104.4 | | | 5608.0 | | | 20998 | | |
| 240281 | | Controls | | | None | | None | | | M | | | 58 | | 4.1 | | | 0.0 | | | 2.3 | | | 4.6 | | | 1599.0 | | | 21698 | | |
| 240299 | | Controls | | | None | | None | | | M | | | 53 | | 2.2 | | | 0.0 | | | 2.5 | | | 12.7 | | | 2243.2 | | | 20880 | | |
| 240340 | | Controls | | | None | | None | | | M | | | 53 | | 0.2 | | | 0.0 | | | 4.3 | | | 11.6 | | | 4844.0 | | | 17379 | | |
| 240422 | | Controls | | | None | | None | | | M | | | 51 | | 1.0 | | | 0.0 | | | 6.1 | | | 4.9 | | | 1829.0 | | | 20510 | | |
| 240423 | | Controls | | | None | | None | | | M | | | 54 | | 2.4 | | | 0.0 | | | 9.8 | | | 86.0 | | | 2589.0 | | | 20468 | | |
| 240430 | | Controls | | | None | | None | | | M | | | 51 | | 2.4 | | | 1.3 | | | 14.4 | | | 53.6 | | | 4337.0 | | | 19259 | | |
| 180541 | | High-risk | | | HBV | | None | | | F | | | 44 | | n/a | | | 1.3 | | | 1.3 | | | 3.9 | | | 2606.7 | | | 21526 | | |
| 190014 | | High-risk | | | HBV | | None | | | M | | | 47 | | 3.4 | | | 0.0 | | | 20.0 | | | 16.9 | | | 3402.5 | | | 20504 | | |
| 190035 | | High-risk | | | HBV | | None | | | M | | | 43 | | n/a | | | 0.0 | | | 4.6 | | | 69.5 | | | 3172.5 | | | 20380 | | |
| 190188 | | High-risk | | | HBV | | None | | | F | | | 54 | | 4.3 | | | 0.0 | | | 1.4 | | | 32.1 | | | 2050.6 | | | 21178 | | |
| 190242 | | High-risk | | | HBV | | None | | | M | | | 29 | | 2.9 | | | 0.0 | | | 5.1 | | | 37.2 | | | 1050.0 | | | 21797 | | |
| 190261 | | High-risk | | | HBV | | None | | | M | | | 51 | | n/a | | | 4.1 | | | 100.2 | | | 169.6 | | | 2713.3 | | | 20672 | | |
| 190283 | | High-risk | | | HBV | | None | | | M | | | 38 | | 2.5 | | | 1.3 | | | 8.6 | | | 13.6 | | | 1965.3 | | | 21508 | | |
| 190314 | | High-risk | | | HBV | | None | | | M | | | 32 | | 2.4 | | | 1.3 | | | 38.0 | | | 94.5 | | | 6094.7 | | | 20200 | | |
| 190374 | | High-risk | | | HBV | | None | | | F | | | 37 | | 2.6 | | | 0.0 | | | 1.4 | | | 6.8 | | | 1387.1 | | | 21651 | | |
| 190433 | | High-risk | | | HBV | | None | | | M | | | 47 | | 3.3 | | | 1.3 | | | 7.3 | | | 6.1 | | | 1066.3 | | | 21723 | | |
| 190531 | | High-risk | | | HBV | | None | | | F | | | 60 | | 2.3 | | | 0.0 | | | 0.0 | | | 4.1 | | | 2744.3 | | | 19199 | | |
| 200107 | | High-risk | | | HBV | | None | | | Fe | | | 43 | | 2.6 | | | 0.0 | | | 4.5 | | | 19.6 | | | 2246.1 | | | 17585 | | |
| 200129 | | High-risk | | | HBV | | None | | | M | | | 27 | | 1.7 | | | 2.6 | | | 2.6 | | | 7.9 | | | 4559.3 | | | 20036 | | |
| 200163 | | High-risk | | | HBV | | None | | | M | | | 58 | | 1.9 | | | 2.3 | | | 37.3 | | | 137.8 | | | 4025.4 | | | 21577 | | |
| 200449 | | High-risk | | | HBV | | None | | | M | | | 58 | | 5.3 | | | 0.0 | | | 9.5 | | | 16.3 | | | 3333.7 | | | 18524 | | |
| 190013 | | High-risk | | | HCV | | None | | | F | | | 56 | | n/a | | | 0.0 | | | 7.3 | | | 30.9 | | | 8990.6 | | | 20133 | | |
| 190017 | | High-risk | | | HCV | | None | | | M | | | 59 | | 2.2 | | | 0.0 | | | 4.8 | | | 9.5 | | | 3617.5 | | | 19994 | | |
| 190049 | | High-risk | | | HCV | | None | | | F | | | 68 | | 3.7 | | | 1.4 | | | 14.4 | | | 27.4 | | | 3850.0 | | | 19350 | | |
| 190089 | | High-risk | | | HCV | | None | | | F | | | 43 | | n/a | | | 0.0 | | | 4.1 | | | 57.2 | | | 2263.3 | | | 20541 | | |
| 190099 | | High-risk | | | HCV | | None | | | M | | | 48 | | 3.0 | | | 0.0 | | | 4.0 | | | 12.0 | | | 1407.8 | | | 20953 | | |
| 190107 | | High-risk | | | HCV | | None | | | F | | | 48 | | 2.4 | | | 1.3 | | | 27.0 | | | 66.1 | | | 3396.7 | | | 20727 | | |
| 190141 | | High-risk | | | HCV | | None | | | F | | | 42 | | 2.3 | | | 0.0 | | | 12.0 | | | 18.1 | | | 1565.9 | | | 19661 | | |
| 190171 | | High-risk | | | HCV | | None | | | F | | | 80 | | 4.3 | | | 1.4 | | | 15.2 | | | 34.5 | | | 2129.4 | | | 21474 | | |
| 190191 | | High-risk | | | HCV | | None | | | M | | | 47 | | 2.5 | | | 3.9 | | | 28.9 | | | 60.6 | | | 5415.8 | | | 20130 | | |
| 190230 | | High-risk | | | HCV | | None | | | M | | | 49 | | n/a | | | 0.0 | | | 10.7 | | | 40.0 | | | 2072.2 | | | 20966 | | |
| 190319 | | High-risk | | | HCV | | None | | | F | | | 61 | | 3.2 | | | 0.0 | | | 2.8 | | | 15.4 | | | 2006.3 | | | 19910 | | |
| 190337 | | High-risk | | | HCV | | None | | | F | | | 60 | | 2.1 | | | 1.3 | | | 25.2 | | | 47.7 | | | 3641.2 | | | 21116 | | |
| 190384 | | High-risk | | | HCV | | None | | | F | | | 53 | | 1.0 | | | 0.0 | | | 12.5 | | | 63.9 | | | 2522.2 | | | 21167 | | |
| 190389 | | High-risk | | | HCV | | None | | | F | | | 35 | | 2.1 | | | 0.0 | | | 2.6 | | | 42.0 | | | 2007.8 | | | 20201 | | |
| 190443 | | High-risk | | | HCV | | None | | | M | | | 71 | | 5.0 | | | 0.0 | | | 1.3 | | | 7.6 | | | 2523.6 | | | 20848 | | |
| 200029 | | High-risk | | | ASH | | None | | | F | | | 60 | | n/a | | | 3.9 | | | 49.8 | | | 120.8 | | | 2820.0 | | | 21343 | | |
| 210230 | | High-risk | | | ASH | | None | | | M | | | 30 | | n/a | | | 0.0 | | | 2.3 | | | 16.2 | | | 1461.0 | | | 21797 | | |
| 210404 | | High-risk | | | ASH | | None | | | M | | | 40 | | 1.7 | | | 0.0 | | | 8.0 | | | 9.0 | | | 2389.0 | | | 21709 | | |
| 210407 | | High-risk | | | ASH | | None | | | M | | | 41 | | 2.4 | | | 0.0 | | | 66.0 | | | 206.0 | | | 4659.0 | | | 21115 | | |
| 210578 | | High-risk | | | ASH | | None | | | M | | | 64 | | 1.5 | | | 0.0 | | | 1.3 | | | 6.2 | | | 1249.5 | | | 21325 | | |
| 210677 | | High-risk | | | ASH | | None | | | M | | | 46 | | 4.3 | | | 1.0 | | | 1.0 | | | 12.0 | | | 1665.0 | | | 20220 | | |
| 220015 | | High-risk | | | ASH | | None | | | M | | | 41 | | n/a | | | 4.4 | | | 12.2 | | | 55.6 | | | 5546.7 | | | 20678 | | |
| 220113 | | High-risk | | | ASH | | None | | | M | | | 49 | | n/a | | | 11.0 | | | 112.0 | | | 515.0 | | | 10915.0 | | | 21238 | | |
| 220116 | | High-risk | | | ASH | | None | | | M | | | 48 | | 2.8 | | | 2.4 | | | 7.4 | | | 4.9 | | | 5943.2 | | | 21530 | | |
| 220382 | | High-risk | | | ASH | | None | | | M | | | 67 | | 7.2 | | | 2.0 | | | 19.0 | | | 78.0 | | | 2172.0 | | | 21342 | | |
| 190151 | | High-risk | | | ASH | | None | | | M | | | 62 | | 4.3 | | | 0.0 | | | 4.3 | | | 18.4 | | | 2080.5 | | | 20889 | | |
| 190256 | | High-risk | | | ASH | | None | | | M | | | 47 | | 2.2 | | | 1.4 | | | 5.7 | | | 17.2 | | | 1475.1 | | | 20630 | | |
| 190271 | | High-risk | | | ASH | | None | | | F | | | 50 | | n/a | | | 0.0 | | | 1.4 | | | 17.8 | | | 1426.3 | | | 21573 | | |
| 190356 | | High-risk | | | ASH | | None | | | M | | | 51 | | n/a | | | 4.1 | | | 49.7 | | | 179.9 | | | 2705.1 | | | 21455 | | |
| 210286 | | High-risk | | | ASH | | None | | | M | | | 62 | | 2.9 | | | 2.5 | | | 48.1 | | | 135.7 | | | 4913.3 | | | 20934 | | |
| 190067 | | High-risk | | | MASLD | | None | | | M | | | 40 | | n/a | | | 0.0 | | | 24.7 | | | 31.8 | | | 1867.1 | | | 21826 | | |
| 190104 | | High-risk | | | MASLD | | None | | | M | | | 33 | | n/a | | | 2.2 | | | 38.9 | | | 148.9 | | | 5955.6 | | | 20308 | | |
| 190196 | | High-risk | | | MASLD | | None | | | M | | | 41 | | n/a | | | 1.4 | | | 9.8 | | | 29.2 | | | 1341.1 | | | 20081 | | |
| 190218 | | High-risk | | | MASLD | | None | | | M | | | 55 | | n/a | | | 0.0 | | | 14.6 | | | 43.8 | | | 3116.5 | | | 20298 | | |
| 190233 | | High-risk | | | MASLD | | None | | | M | | | 41 | | n/a | | | 3.9 | | | 16.7 | | | 65.9 | | | 6774.7 | | | 20525 | | |
| 190289 | | High-risk | | | MASLD | | None | | | F | | | 48 | | n/a | | | 0.0 | | | 5.1 | | | 30.3 | | | 2678.9 | | | 20965 | | |
| 190434 | | High-risk | | | MASLD | | None | | | F | | | 40 | | n/a | | | 2.1 | | | 10.5 | | | 58.9 | | | 3174.7 | | | 21007 | | |
| 190699 | | High-risk | | | MASLD | | None | | | F | | | 57 | | n/a | | | 1.3 | | | 1.3 | | | 38.9 | | | 2567.4 | | | 21084 | | |
| 190750 | | High-risk | | | MASLD | | None | | | M | | | 58 | | n/a | | | 11.9 | | | 81.3 | | | 231.4 | | | 3936.0 | | | 21075 | | |
| 200040 | | High-risk | | | MASLD | | None | | | M | | | 63 | | n/a | | | 4.2 | | | 13.7 | | | 40.0 | | | 5289.5 | | | 21420 | | |
| 200155 | | High-risk | | | MASLD | | None | | | M | | | 42 | | n/a | | | 0.0 | | | 16.2 | | | 41.2 | | | 3425.0 | | | 21241 | | |
| 200646 | | High-risk | | | MASLD | | None | | | F | | | 61 | | n/a | | | 20.2 | | | 167.1 | | | 667.7 | | | 5589.9 | | | 21150 | | |
| 210035 | | High-risk | | | MASLD | | None | | | M | | | 33 | | n/a | | | 1.2 | | | 23.9 | | | 94.7 | | | 4544.2 | | | 21028 | | |
| 210151 | | High-risk | | | MASLD | | None | | | M | | | 34 | | n/a | | | 1.3 | | | 0.0 | | | 5.2 | | | 3929.8 | | | 21494 | | |
| 210203 | | High-risk | | | MASLD | | None | | | M | | | 23 | | n/a | | | 3.8 | | | 32.6 | | | 256.7 | | | 6337.2 | | | 20096 | | |
| 190669 | | HCC | | | HBV/Cir | | 0 | | | M | | | 62 | | 26.2 | | | 0.0 | | | 4.0 | | | 9.3 | | | 3741.1 | | | 21002 | | |
| 190698 | | HCC | | | HBV/Cir | | 0 | | | F | | | 56 | | 7.0 | | | 16.5 | | | 10.5 | | | 40.4 | | | 7247.5 | | | 21035 | | |
| 190713 | | HCC | | | HBV/Cir | | 0 | | | M | | | 67 | | 12.0 | | | 3.0 | | | 19.8 | | | 48.6 | | | 9748.8 | | | 20734 | | |
| 190752 | | HCC | | | Cir | | 0 | | | F | | | 69 | | 0.7 | | | 109.4 | | | 1321.2 | | | 1594.6 | | | 26224.7 | | | 21944 | | |
| 190789 | | HCC | | | HBV/Cir | | 0 | | | M | | | 49 | | 38.4 | | | 18.8 | | | 341.9 | | | 416.4 | | | 60221.1 | | | 20893 | | |
| 190804 | | HCC | | | HBV/Cir | | 0 | | | F | | | 49 | | 61.5 | | | 10.1 | | | 50.7 | | | 299.4 | | | 31121.9 | | | 20904 | | |
| 190833 | | HCC | | | HBV | | 0 | | | M | | | 60 | | 4.6 | | | 53.6 | | | 418.5 | | | 1130.5 | | | 39930.6 | | | 21750 | | |
| 200020 | | HCC | | | ASH/Cir | | A | | | M | | | 63 | | 14.8 | | | 1.3 | | | 11.3 | | | 21.3 | | | 56226.5 | | | 20095 | | |
| 200121 | | HCC | | | HBV/Cir | | 0 | | | M | | | 59 | | 3.0 | | | 0.0 | | | 6.4 | | | 7.7 | | | 5075.8 | | | 19528 | | |
| 200355 | | HCC | | | ASH/Cir | | 0 | | | M | | | 66 | | 7.3 | | | 1.4 | | | 4.1 | | | 4.1 | | | 1547.3 | | | 19311 | | |
| 200368 | | HCC | | | HBV | | 0 | | | M | | | 60 | | 35.5 | | | 0.0 | | | 5.2 | | | 9.1 | | | 1174.5 | | | 20330 | | |
| 200519 | | HCC | | | HCV/Cir | | 0 | | | M | | | 46 | | 545.2 | | | 4.8 | | | 28.5 | | | 67.8 | | | 2501.6 | | | 21182 | | |
| 200520 | | HCC | | | HBV/Cir | | 0 | | | M | | | 49 | | 327.1 | | | 3.5 | | | 15.0 | | | 57.9 | | | 3870.5 | | | 21760 | | |
| 200537 | | HCC | | | ASH/Cir | | 0 | | | M | | | 59 | | 13.0 | | | 1.3 | | | 14.1 | | | 27.0 | | | 7234.3 | | | 19567 | | |
| 200597 | | HCC | | | Cir | | 0 | | | M | | | 78 | | 4.9 | | | 6.2 | | | 6.2 | | | 4.9 | | | 2502.0 | | | 21430 | | |
| 200620 | | HCC | | | HCV/Cir | | 0 | | | M | | | 65 | | 10.1 | | | 80.4 | | | 508.5 | | | 701.7 | | | 10823.2 | | | 21452 | | |
| 200621 | | HCC | | | HBV/Cir | | 0 | | | M | | | 67 | | 2.4 | | | 100.6 | | | 941.1 | | | 2165.5 | | | 44622.5 | | | 21625 | | |
| 200623 | | HCC | | | HCV/Cir | | 0 | | | F | | | 65 | | 201.4 | | | 3.7 | | | 3.7 | | | 11.0 | | | 7275.9 | | | 20653 | | |
| 190681 | | HCC | | | HBV/Cir | | A | | | M | | | 66 | | 8.5 | | | 64.2 | | | 19.1 | | | 68.3 | | | 2277.8 | | | 20481 | | |
| 200044 | | HCC | | | HBV | | A | | | M | | | 56 | | 5.2 | | | 64.3 | | | 630.3 | | | 1065.3 | | | 14274.7 | | | 20619 | | |
| 200078 | | HCC | | | HBV/Cir | | A | | | M | | | 69 | | 2.3 | | | 1.3 | | | 3.8 | | | 6.2 | | | 1151.6 | | | 21250 | | |
| 200161 | | HCC | | | HBV/Cir | | A | | | M | | | 48 | | 2755.0 | | | 49.4 | | | 52.2 | | | 86.1 | | | 11167.1 | | | 21000 | | |
| 200181 | | HCC | | | HBV/Cir | | A | | | M | | | 62 | | 3.2 | | | 162.4 | | | 1032.6 | | | 1695.7 | | | 65013.7 | | | 20604 | | |
| 200189 | | HCC | | | n/a | | A | | | M | | | 68 | | 3.3 | | | 1.3 | | | 3.8 | | | 8.9 | | | 4033.6 | | | 20823 | | |
| 200211 | | HCC | | | ASH | | A | | | M | | | 73 | | 5.1 | | | 65.1 | | | 589.1 | | | 575.0 | | | 17429.2 | | | 21182 | | |
| 200225 | | HCC | | | n/a | | A | | | M | | | 71 | | 2.1 | | | 109.1 | | | 1108.8 | | | 2573.7 | | | 35735.1 | | | 21316 | | |
| 200287 | | HCC | | | HCV/Cir | | A | | | F | | | 62 | | 84.4 | | | 47.2 | | | 608.1 | | | 1075.5 | | | 58764.9 | | | 20812 | | |
| 200328 | | HCC | | | HBV/Cir | | A | | | M | | | 52 | | 2.2 | | | 0.0 | | | 1.3 | | | 10.1 | | | 2411.3 | | | 20923 | | |
| 200453 | | HCC | | | Cir | | A | | | M | | | 82 | | 4.6 | | | 58.0 | | | 49.0 | | | 45.1 | | | 3579.9 | | | 20570 | | |
| 200476 | | HCC | | | HBV/Cir | | A | | | F | | | 45 | | 212.7 | | | 2.4 | | | 4.8 | | | 9.5 | | | 784.6 | | | 21169 | | |
| 200496 | | HCC | | | Cir | | A | | | M | | | 52 | | 3.5 | | | 2.9 | | | 25.7 | | | 38.6 | | | 2238.9 | | | 17604 | | |
| 200642 | | HCC | | | ASH/Cir | | A | | | M | | | 74 | | 2.8 | | | 1547.1 | | | 947.8 | | | 1701.5 | | | 8234.0 | | | 17692 | | |
| 210155 | | HCC | | | HBV/Cir | | 0 | | | M | | | 56 | | 149.0 | | | 67.4 | | | 42.3 | | | 58.1 | | | 10389.8 | | | 20066 | | |
| 210184 | | HCC | | | ASH/Cir | | A | | | M | | | 76 | | 14.1 | | | 4.9 | | | 21.0 | | | 87.9 | | | 4975.4 | | | 20352 | | |
| 210255 | | HCC | | | HBV/Cir | | A | | | M | | | 56 | | 17.6 | | | 21.4 | | | 12.0 | | | 38.7 | | | 1742.2 | | | 18861 | | |
| 210393 | | HCC | | | HBV/Cir | | A | | | M | | | 46 | | 12.6 | | | 6.6 | | | 32.8 | | | 53.8 | | | 2318.8 | | | 19182 | | |
| 190680 | | HCC | | | ASH/Cir | | B | | | M | | | 52 | | 4.3 | | | 1.4 | | | 13.9 | | | 19.4 | | | 4395.3 | | | 21302 | | |
| 190710 | | HCC | | | HBV/Cir | | A | | | M | | | 67 | | 21.2 | | | 10.4 | | | 19.4 | | | 11.9 | | | 2893.8 | | | 21134 | | |
| 190751 | | HCC | | | HBV/Cir | | A | | | M | | | 45 | | 4.1 | | | 8.8 | | | 75.8 | | | 342.4 | | | 15811.1 | | | 19217 | | |
| 190849 | | HCC | | | HBV | | A | | | M | | | 49 | | 394.6 | | | 27.1 | | | 269.2 | | | 766.4 | | | 10624.4 | | | 21704 | | |
| 200025 | | HCC | | | ASH/Cir | | C | | | M | | | 58 | | 4.4 | | | 66.2 | | | 29.8 | | | 98.8 | | | 2524.4 | | | 20693 | | |
| 200248 | | HCC | | | ASH/Cir | | B | | | M | | | 46 | | 20.1 | | | 5.9 | | | 108.3 | | | 309.9 | | | 10026.2 | | | 16988 | | |
| 200272 | | HCC | | | HBV/Cir | | A | | | M | | | 48 | | 1191.0 | | | 13.9 | | | 8.4 | | | 20.9 | | | 573.3 | | | 19003 | | |
| 200326 | | HCC | | | HCV/Cir | | A | | | M | | | 77 | | 121.8 | | | 3.8 | | | 15.2 | | | 60.7 | | | 2890.9 | | | 20973 | | |
| 200331 | | HCC | | | HBV/Cir | | B | | | M | | | 73 | | 50073 | | | 2.7 | | | 61.7 | | | 37.0 | | | 2169.7 | | | 20406 | | |
| 200345 | | HCC | | | HBV/Cir | | B | | | M | | | 49 | | 2.4 | | | 34.5 | | | 15.4 | | | 46.4 | | | 932.6 | | | 21185 | | |
| 200467 | | HCC | | | ASH | | B | | | M | | | 86 | | 1.7 | | | 509.5 | | | 345.5 | | | 887.1 | | | 6868.8 | | | 20519 | | |
| 200488 | | HCC | | | HBV/Cir | | B | | | M | | | 65 | | 148.6 | | | 20.2 | | | 16.1 | | | 51.2 | | | 1959.0 | | | 19690 | | |
| 190688 | | HCC | | | HBV/Cir | | C | | | M | | | 58 | | 3.6 | | | 16210.3 | | | 2718.4 | | | 7294.1 | | | 20031.1 | | | 20729 | | |
| 200008 | | HCC | | | HBV/Cir | | C | | | M | | | 51 | | 916.8 | | | 12587.1 | | | 4321.8 | | | 4080.2 | | | 65772.9 | | | 20764 | | |
| 200022 | | HCC | | | ASH/Cir | | C | | | M | | | 47 | | 6828.0 | | | 73914.0 | | | 18428.9 | | | 51829.3 | | | 72942.4 | | | 19295 | | |
| 200066 | | HCC | | | HBV | | C | | | M | | | 46 | | 1260.0 | | | 0.0 | | | 54.1 | | | 127.3 | | | 2058.9 | | | 19613 | | |
| 200079 | | HCC | | | HBV/Cir | | C | | | M | | | 52 | | 5712.0 | | | 258.0 | | | 138.0 | | | 405.4 | | | 7762.2 | | | 21112 | | |
| 200109 | | HCC | | | Cir | | C | | | F | | | 62 | | 5868.0 | | | 105.1 | | | 30.7 | | | 51.2 | | | 3852.4 | | | 19664 | | |
| 200251 | | HCC | | | ASH/Cir | | C | | | M | | | 52 | | 4.1 | | | 175.1 | | | 83.8 | | | 377.3 | | | 12976.1 | | | 20758 | | |
| 200284 | | HCC | | | HBV | | C | | | F | | | 64 | | 48.9 | | | 6.8 | | | 20.4 | | | 89.9 | | | 10495.6 | | | 19473 | | |
| 200577 | | HCC | | | n/a | | C | | | M | | | 74 | | 13.5 | | | 145.0 | | | 65.7 | | | 271.2 | | | 2460.5 | | | 20703 | | |
| 210259 | | HCC | | | HCV/Cir | | C | | | M | | | 71 | | 29.2 | | | 19.9 | | | 23.7 | | | 32.4 | | | 894.3 | | | 20187 | | |
| 210266 | | HCC | | | HBV/Cir | | C | | | M | | | 57 | | 17.5 | | | 24.0 | | | 31.5 | | | 70.7 | | | 730.5 | | | 19950 | | |
| 210305 | | HCC | | | HBV/Cir | | C | | | M | | | 56 | | 54.6 | | | 2680.6 | | | 577.9 | | | 1816.1 | | | 7866.8 | | | 20428 | | |
| HCC, hepatocellular carcinoma; HBV, hepatitis B virus; HCV, hepatitis C virus; ASH, Alcoholic steatohepatitis; MASLD, Metabolic dysfunction-associated steatotic liver disease; Cir, cirrhosis; BCLC, Barcelona Clinic Liver Cancer; n/a, not applicable | | | | | | | | | | | | | | | | | | | | | | | | | | | | | | | | |

**DIGITAL MIQE CHECKLIST**

| **Item to Check** | **Provided** | **Comment** |
| --- | --- | --- |
| **1. SPECIMEN** |  |  |
| Detailed description of specimen type and numbers | **Y** | Explained in **Materials and Methods**, **Table 1** |
| Sampling procedure (including time to storage) | **Y** | Explained in **Materials and** **Methods** |
| Sample aliquotation, storage conditions and duration | **Y** | Explained in **Materials and** **Methods** |
| **2. NUCLEIC ACID EXTRACTION** |  |  |
| Description of extraction method including amount of sample processed | **Y** | Explained in **Materials and** **Methods** |
| Volume of solvent used to elute/resuspend extract | **Y** | Explained in **Materials and** **Methods** |
| Number of extraction replicates | **N** | Used up all of samples |
| Extraction blanks included? | **Y** | Explained in **Materials and** **Methods** (no-template control) |
| **3. NUCLEIC ACID ASSESSMENT AND STORAGE** |  |  |
| Method to evaluate quality of nucleic acids | **Y** | Explained in **Materials and Methods** (evaluation by NanoDrop spectrophotometer) |
| Method to evaluate quantity of nucleic acids (including molecular weight and calculations when using mass) | **Y** | Explained in **Materials and Methods** (quantification using NanoDrop spectrophotometer) |
| Storage conditions: temperature, concentration, duration, buffer, aliquots | **Y** | Explained in **Materials and Methods** (tested approximately after DNA extraction) |
| Clear description of dilution steps used to prepare working DNA solution | **N** | All samples used up |
| **4. NUCLEIC ACID MODIFICATION** |  |  |
| Template modification (digestion, sonication, pre-amplification, bisulphite etc.) | **Y** | Explained in **Materials and Methods** (bisulfite treatment) |
| Details of repurification following modification if performed | **Y** | Explained in **Materials and Methods** |
| **5. REVERSE TRANSCRIPTION** |  |  |
| cDNA priming method and concentration | **N** | Not applicable |
| One or two step protocol (include reaction details for two step) | **N** | Not applicable |
| Amount of RNA added per reaction | **N** | Not applicable |
| Detailed reaction components and conditions | **N** | Not applicable |
| Estimated copies measured with and without addition of RT* | **N** | Not applicable |
| Manufacturer of reagents used with catalogue and lot numbers | **N** | Not applicable |
| Storage of cDNA: temperature, concentration, duration, buffer and aliquots | **N** | Not applicable |
| **6. dPCR OLIGONUCLEOTIDES DESIGN AND TARGET INFORMATION** |  |  |
| Sequence accession number or official gene symbol | **Y** | Explained in **Supplemental Table S2** |
| Method (software) used for design and *in silico* verification | **N** | Not explained in the manuscript, but IDT website was utilized for *in silico* study |
| Location of amplicon | **Y** | Explained in **Supplemental Table S2** |
| Amplicon length | **Y** | Explained in **Supplemental Table S2** |
| Primer and probe sequences (or amplicon context sequence)** | **Y** | Explained in **Supplementary Table S2** (amplicon context regions) |
| Location and identity of any modifications | **N** | Not applicable |
| Manufacturer of oligonucleotides | **Y** | Explained in **Materials and Methods** |
| **7. dPCR PROTOCOL** |  |  |
| Manufacturer of dPCR instrument and instrument model | **Y** | Explained in **Materials and Methods** |
| Buffer/kit manufacturer with catalogue and lot number | **Y** | Explained in **Materials and** **Methods** |
| Primer and probe concentration | **N** | Not explained in the manuscript, due to manufacturer's disclosure decision (100-400 nM) |
| Pre-reaction volume and composition (incl. amount of template and if restriction enzyme added) | **N** | Not applicable |
| Template treatment (initial heating or chemical denaturation) | **N** | Not applicable |
| Polymerase identity and concentration, Mg++ and dNTP concentrations*** | **Y** | Explained in **Materials and Methods** (PCR master mix was provided by manufacturer) |
| Complete thermocycling parameters | **Y** | Explained in **Materials and** **Methods** |
| **8. ASSAY VALIDATION** |  |  |
| Details of optimisation performed | **Y** | Explained in **Materials and** **Methods** |
| Analytical specificity (vs. related sequences) and limit of blank (LOB) | **N** | Not explained in the manuscript, but analytical specificity tests, including cross-reactivity and LOB, were completed. The LOB was 2.6 copies and no cross-reactivity was observed. |
| Analytical sensitivity/LoD and how this was evaluated | **N** | Not explained in the manuscript, but analytical sensitivity tests including LOD and linearity, were completed. The LOD was found to be 12.5 pg/mL, with linearity confirmed across the 0-5 ng/mL (R^2^ = 0.999). |
| Testing for inhibitors (from biological matrix/extraction) | **N** | Not explained in the manuscript, but an interference test was completed. No interference was observed in substances such as hemoglobin, cholesterol, and albumin). |
| **9. DATA ANALYSIS** |  |  |
| Description of dPCR experimental design | **Y** | Explained in **Materials and** **Methods** |
| Comprehensive details negative and positive of controls (whether applied for QC or for estimation of error) | **Y** | Explained in **Materials and** **Methods** (commercial standards) |
| Partition classification method (thresholding) | **Y** | Explained in **Materials and** **Methods** (auto-threshold) |
| Examples of positive and negative experimental results (including fluorescence plots in supplemental material) | **N** | Not explained in the manuscript, plan to submit if required |
| Description of technical replication | **N** | Not applicable |
| Repeatability (intra-experiment variation) | **N** | Not explained in the manuscript, but a repeatability test was completed with a coefficient deviation of less than 20%. |
| Reproducibility (inter-experiment/user/lab etc. variation) | **N** | Not explained in the manuscript, but a reproducibility test was completed with a coefficient deviation of less than 20%. |
| Number of partitions measured (average and standard deviation) | **Y** | Not explained in the manuscript (Average ± SD, 20635.7±1417.8 in the validation study) |
| Partition volume | **N** | Not applicable |
| Copies per partition (λ or equivalent) (average and standard deviation) | **N** | Not applicable |
| dPCR analysis program (source, version) | **Y** | Explained in **Materials and** **Methods** |
| Description of normalisation method | **Y** | Explained in **Materials and** **Methods** |
| Statistical methods used for analysis | **Y** | Explained in **Materials and** **Methods** |
| Data transparency | **Y** | Available on request if corresponding author has given permission |
